# Ecological inducers of the yeast filamentous growth pathway reveal environment-dependent roles for pathway components

**DOI:** 10.1128/msphere.00284-23

**Published:** 2023-09-21

**Authors:** Matthew D. Vandermeulen, Paul J. Cullen

**Affiliations:** 1 Department of Biological Sciences, University at Buffalo, Buffalo, New York, USA; University of Georgia, Athens, Georgia, USA

**Keywords:** MAPK, signaling, filamentous growth, yeast ecology, evolution, pectin, ethanol, galactose

## Abstract

Signaling modules, such as mitogen-activated protein kinase (MAPK) pathways, are evolutionarily conserved drivers of cell differentiation and stress responses. In many fungal species including pathogens, MAPK pathways control filamentous growth, where cells differentiate into an elongated cell type. The convenient model budding yeast *Saccharomyces cerevisiae* undergoes filamentous growth by the filamentous growth (fMAPK) pathway; however, the inducers of the pathway remain unclear, perhaps because pathway activity has been mainly studied in laboratory conditions. To address this knowledge gap, an ecological framework was used, which uncovered new fMAPK pathway inducers, including pectin, a material found in plants, and the metabolic byproduct ethanol. We also show that induction by a known inducer of the pathway, the non-preferred carbon source galactose, required galactose metabolism and induced the pathway differently than glucose limitation or other non-preferred carbon sources. By exploring fMAPK pathway function in fruit, we found that induction of the pathway led to visible digestion of fruit rind through a known target, *PGU1*, which encodes a pectolytic enzyme. Combinations of inducers (galactose and ethanol) stimulated the pathway to near-maximal levels, which showed dispensability of several fMAPK pathway components (e.g., mucin sensor, p21-activated kinase), but not others (e.g., adaptor, MAPKKK) and required the Ras2-protein kinase A pathway. This included a difference between the transcription factor binding partners for the pathway, as Tec1p, but not Ste12p, was partly dispensable for fMAPK pathway activity. Thus, by exploring ecologically relevant stimuli, new modes of MAPK pathway signaling were uncovered, perhaps revealing how a pathway can respond differently to specific environments.

**IMPORTANCE:**

Filamentous growth is a cell differentiation response and important aspect of fungal biology. In plant and animal fungal pathogens, filamentous growth contributes to virulence. One signaling pathway that regulates filamentous growth is an evolutionarily conserved MAPK pathway. The yeast *Saccharomyces cerevisiae* is a convenient model to study MAPK-dependent regulation of filamentous growth, although the inducers of the pathway are not clear. Here, we exposed yeast cells to ecologically relevant compounds (e.g., plant compounds), which identified new inducers of the MAPK pathway. In combination, the inducers activated the pathway to near-maximal levels but did not cause detrimental phenotypes associated with previously identified hyperactive alleles. This context allowed us to identify conditional bypass for multiple pathway components. Thus, near-maximal induction of a MAPK pathway by ecologically relevant inducers provides a powerful tool to assess cellular signaling during a fungal differentiation response.

## INTRODUCTION

Organisms can sense and respond to signals in the environment. One way this occurs is by signal transduction pathways, such as evolutionarily conserved mitogen-activated protein kinase (MAPK) pathways (1 – 3). MAPK pathways sense and relay the signals from external/internal environments to induce a response, which typically occurs by the induction of gene expression (4 – 8). Much interest surrounding MAPK pathways comes from studies of their mis-regulation in diseases like cancer (9 – 11). Some aspects of MAPK pathways remain poorly defined, and the stimuli that trigger pathways remain in many cases mysterious.

In addition to their role in animals, MAPK pathways also regulate biological responses in plants (12, 13) and fungi (14 – 16). In fungi, including single-celled yeasts, MAPK pathways can promote a cell differentiation response called filamentous growth. In pathogenic yeast, like the human pathogen *Candida albicans* (17, 18), and the plant pathogen *Ustilago maydis* (19), filamentous growth is controlled by MAPK pathways and is critical for virulence. During filamentous growth, cells grow in elongated structures (i.e., hyphae or pseudohyphae), and express specific adhesion molecules which promote attachment to surfaces and invasion into the host. In plant fungal pathogens (20 – 23), MAPK pathways respond to cues from the plant surface to initiate invasion (24, 25). Although intensively studied, how fungal cells recognize, attach, and invade diverse environments through the action of signaling pathways remains an open question.

**Fig 1:**
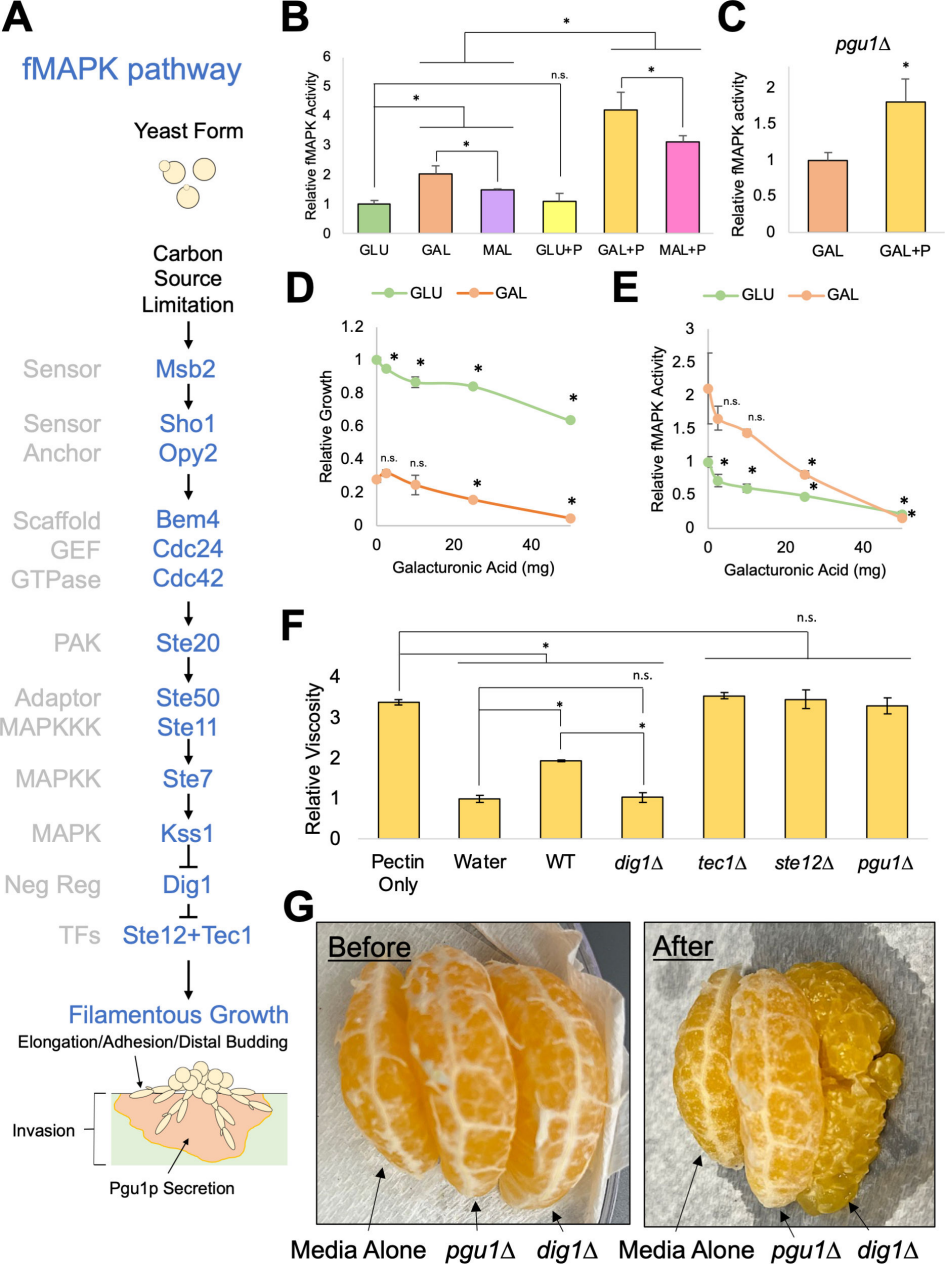
The fMAPK pathway is induced by pectin and regulates pectin breakdown in fruit. (**A**) The fMAPK pathway regulates the switch to filamentous growth upon carbon source limitation. (**B**) β-galactosidase (*FRE-lacZ*) assays. Wild-type cells (PC313) were grown for 24 h in 5 mL of synthetic media with indicated carbon source. +P = + 1% pectin. Average relative fMAPK pathway activity for at least three replicates is reported, with values for glucose (GLU) set to 1. Error bars represent standard deviation. *, *P*-value <0.05 by Student’s *t*-test compared to the indicated condition. (**C**) Same experiment as panel B except in the *pgu1*∆ mutant (PC7833). (**D**) Relative growth of wild-type cells (PC313) in 5 mL synthetic media with the indicated carbon source after growth for 18 h. Galacturonic acid was added as indicated (milligrams). Average relative growth determined by optical density at 600 nm (OD_600_) across at least three replicates is reported, with values for GLU set to 1. Error bars represent standard deviation. *, *P*-value <0.05 by Student’s *t*-test comparing galacturonic acid to carbon source alone. (**E**) Same as panel D except β-galactosidase (*FRE-lacZ*) assay for relative fMAPK pathway activity. (**F**) Viscosity assay. Wild-type (WT) cells (PC313) and the *pgu1*∆ (PC7833), *tec1*∆ (PC7675), *ste12*∆ (PC5651), and *dig1*∆ (PC7676) mutants were grown in GAL + P (1% pectin) solution for 17 h. Tube sedimentation was measured and compared to cultures of water and GAL + P with no cells added (pectin only) as controls for pectin breakdown and no pectin breakdown, respectively. Average relative viscosity from three replicates are reported, with values of tube sedimentation in water set to 1. Error bars represent standard deviation. *, *P*-value <0.05 by Student’s *t*-test compared to indicated condition/strain. (**G**) Pectin digestion. Mandarin orange wedges were incubated in supernatants of *pgu1*∆ and *dig1*∆ mutants for 24 h, which were derived from a 24-h growth culture of cells in YPGAL, or YPGAL alone as a control. Left, wedges before incubation. Right, wedges after incubation.

The regulatory pathways that control filamentous growth are evolutionarily conserved between fungal species (26, 27). The model organism and saprotrophic budding yeast *Saccharomyces cerevisiae* undergoes filamentous growth (28) and has emerged as a model because of the ease of genetic manipulation and many tools available. In *S. cerevisiae* (28 – 30) and *C. albicans* (18, 31, 32), filamentous growth occurs when cells encounter nutrient limitation. For example, nitrogen limitation (28, 33 – 35) and glucose limitation (29, 30, 36, 37) stimulate filamentous growth in *S. cerevisiae*. Quorum-sensing molecules, which are indicators of cell density (38, 39), also influence filamentous growth and include alcohols like ethanol and butanol in *S. cerevisiae* (40 – 43) and farnesol and tyrosol in *C. albicans* (38, 44). *S. cerevisiae* does not form true hyphae but undergoes a change in morphology from round yeast-form cells to adhesion-linked “chains” of elongated cells that can invade into surfaces (i.e., invasive growth), presumably as a scavenging response (Fig. 1A) (28, 29, 45). Also, *S. cerevisiae* (46 – 48), like *U. maydis* and many other plant-associated fungi (49 – 51), secretes a pectinase enzyme (Pgu1p in *S. cerevisiae*, an endo-polygalacturonnase [52]) during filamentous growth to break down pectin (Fig. 1A), which is a major component of plant cell walls (53 – 56).

Among several MAPK pathways in yeast (57 – 59), the filamentous growth MAPK (fMAPK) pathway is a main regulatory pathway of filamentous growth (28, 35, 60 – 62) (Fig. 1A). The fMAPK pathway is headed by Msb2p, a member of the mucin family of glycoproteins (63 – 68). Msb2p interacts with the tetraspan sensor, Sho1p (67, 69 – 73), and a single pass transmembrane protein, Opy2p, that anchors cytosolic proteins to the plasma membrane (70, 74, 75). Msb2p and Sho1p control the activity of the guanine nucleotide exchange factor Cdc24p, which is the main activator (76 – 78) of the Rho GTPase Cdc42p (79 – 81). Activation of the Cdc42p module requires the bud-site GTPase Rsr1p (82), the polarity adaptor Bem1p (83 – 85), and the scaffold protein Bem4p (83, 86). Once activated, Cdc42p binds to and activates the p21-activated kinase (PAK), Ste20p (73, 87 – 89). Ste20p itself phosphorylates the MAPKKK, Ste11p, which is recruited to the plasma membrane by Opy2p and another adaptor Ste50p (90, 91). At the plasma membrane, Ste11p phosphorylates the MAPKK, Ste7p, which then phosphorylates the MAPK, Kss1p (87, 92 – 95). The MAP kinase Kss1p regulates the transcription factors, Ste12p and Tec1p (92, 96 – 99), which dimerize to co-regulate target genes. Ste12p and Tec1p also work with other transcriptional regulators, including the co-activators Msa1p and Msa2p (98), and the strong transcriptional repressor, Dig1p (93, 94, 100).

Unlike pathogens, where ecology in the host is typically considered, studies of the fMAPK pathway in *S. cerevisiae* have mainly been performed in laboratory conditions. However, *Saccharomyces* yeast are commonly found in wild and domesticated habitats, including fruits, tree sap and bark, insect vectors, leaf litter, soil, and rotten wood (101 – 112). Signaling pathways in *S. cerevisiae* may have evolved to sense and respond to stimuli from these diverse environments. Here, we investigated fMAPK pathway activation by compounds expected to be encountered in the wild. We considered plant-associated compounds, like pectin, and the metabolic byproduct and quorum-sensing molecule, ethanol, which is an inhibitor of microbial competitors (113, 114) and an attractant for insect vectors (109, 115 – 118). We also examined carbon sources like galactose more closely, which is abundant in natural habitats of *S. cerevisiae* (108, 109, 119), including forest leaf litter/soil (111, 120), and certain fruits (121 – 123).

This “ecological approach” uncovered new inducers of the fMAPK pathway, including pectin and ethanol. We also found that galactose induced fMAPK pathway activity differently than glucose limitation or other non-preferred carbon sources. Combinations of inducers (galactose with ethanol) activated the pathway to near-maximal levels, which has not been previously observed in laboratory settings. Maximal activation of the fMAPK pathway partly bypassed the requirement of several core components of the pathway (Msb2p, Sho1p, Opy2p, Ste20p, Bem4p, and Tec1p) but not others (Ste50p, Ste11p, and Ste12p). We also identified a critical role for the Ras2-protein kinase A (Ras-PKA) pathway, a known regulator of filamentous growth and the fMAPK pathway (28, 124 – 129), in fMAPK pathway regulation in response to ethanol. Thus, studying a model organism from an ecological perspective provides insights into pathway regulation that may apply to other systems, like pathogens who thrive in the unique ecologies of their hosts.

## RESULTS

### Pectin is a new inducer of the fMAPK pathway

The fMAPK pathway regulates pectinase levels (48); therefore, we tested whether the fMAPK pathway is activated when cells encounter plant material/compounds to promote pectin breakdown. Several plant compounds were tested, including pectin, breakdown products of pectin (di-galacturonic acid and galacturonic acid), and indoleacetic acid (IAA), a plant hormone previously shown to stimulate filamentous growth (130). fMAPK pathway activity was measured in a filamentous strain (Σ1278b background) by a transcriptional reporter (p*FRE-lacZ* [131]). To test the effect of pectin on fMAPK pathway activity, a 1% pectin solution was made in media containing the preferred carbon source glucose, or non-preferred carbon sources, galactose or maltose. In glucose, the activity of the fMAPK pathway was not stimulated by pectin (Fig. 1B, compare GLU to GLU + P). However, pectin stimulated the fMAPK pathway in media supplemented with galactose or maltose (Fig. 1B, compare GAL to GAL + P and MAL to MAL + P). The fact that pectin induced the fMAPK pathway only in the absence of glucose may suggest that pectin induction is subject to glucose repression (GR) (132).

Pectin is a polymer of galacturonic acid and other sugars (55). Pectin and its breakdown product by the Pgu1p enzyme, i.e. di-galacturonic acid, both regulate *PGU1* expression (133, 134); therefore, pectin may be recognized by the fMAPK pathway as a polymer or by its breakdown products. Unlike pectin, di-galacturonic acid did not induce the fMAPK pathway (Fig. S1A). Therefore, di-galacturonic acid may regulate *PGU1* expression through another pathway (Fig. S1B) (132, 133). In cells lacking pectinase (*pgu1*∆), where pectin breakdown does not occur (see below, viscosity assay), pectin induced the fMAPK pathway (Fig. 1C, *pgu1*∆ mutant shows similar increase between GAL and GAL + P as wild type (WT) in Fig. 1B). Thus, the fMAPK pathway is induced by pectin and does not require breakdown products of Pgu1p activity (Fig. S1B).

Presumably, *S. cerevisiae* does not break down di-galacturonic acid into galacturonic acid because galacturonic acid inhibits growth (135). Some fungal and bacterial organisms other than *S. cerevisiae* can break down pectin to galacturonic acid to use as a carbon source (136 – 139). This body of data suggests that galacturonic acid could possibly be encountered in the wild. As expected, galacturonic acid inhibited the growth of yeast cells (Fig. 1D); however, it also caused a reduction in fMAPK pathway activity (Fig. 1E). The reduced fMAPK pathway activity may not be due to growth inhibition, as growth inhibition by other compounds did not cause a reduction in fMAPK pathway activity (see below, ethanol). The plant hormone, IAA, did not affect fMAPK pathway activity (Fig. S1C), although we did not test the carbon source xylose, which induces IAA-dependent invasive growth (130). Collectively, these experiments identify galacturonic acid as an inhibitor and pectin as a new inducer of the fMAPK pathway.

### A function for the fMAPK pathway in pectin degradation in fruit

Yeast cells may break down pectin to improve accessibility to plant tissues, and therefore nutrients, as has been suggested (46 – 48, 140). Pectin may also be broken down to use as a carbon source. This latter possibility seems unlikely because *S. cerevisiae* did not grow in pectin as the sole carbon source (WT optical density at 600 nm (OD_600_) in synthetic medium with 2% pectin as carbon source remained <0.08 after 16 h). Therefore, we focused on testing whether pectin breakdown might improve accessibility to the plant environment.

Pectin breakdown by Pgu1p has been visualized by a plate-based test that measures enzymatic activity (48, 141). To more directly test how pectin breakdown affects accessibility to the plant environment, two tests were developed. The first test was based on the fact that pectin-rich solutions are viscous, which reflects pectin acting as a physical barrier to yeast cells; therefore, we measured changes in viscosity of pectin solutions after incubation with wild-type cells and fMAPK pathway mutants. Viscosity can be measured by determining the time for a weight to reach the bottom of a solution. The viscosity of a 1% pectin solution was measured as a control (Fig. 1F, pectin only) and compared to water, a control for complete pectin breakdown (Fig. 1F, water). The viscosity of a pectin solution was found to be reduced after a 17-h incubation with wild-type cells (Fig. 1F, WT). The reduction in viscosity was dependent on pectinase activity, as seen in cells lacking Pgu1p (Fig. 1F, *pgu1*Δ). The reduction in viscosity was also dependent on the fMAPK pathway as seen in cells lacking transcription factors Tec1p or Ste12p (Fig. 1F, *ste12*Δ or *tec1*Δ). Viscosity was strongly reduced when incubated with cells lacking the negative regulator Dig1p, which has elevated fMAPK pathway activity (Fig. 1F, *dig1*∆). Therefore, the fMAPK pathway functions to reduce viscosity of pectin-based solutions, and may facilitate accessibility to plant-based environments.

In the second test, because *S. cerevisiae* has been isolated on fruit (108, 119), pectin breakdown was examined in rinds of mandarin oranges. These rinds contain a white pith material that is made primarily of pectin among other compounds (142 – 144) (Fig. 1G, before). Mandarin orange wedges were incubated for 24 h in supernatants derived from the *dig1*∆ and *pgu1*∆ mutants and compared to incubation with medium alone (Fig. 1G, images of peels and more images of wedges are shown in Fig. S2). This result showed that the fMAPK pathway and Pgu1p cause a detectable loss of pith material in the fruit rind. Moreover, it was clear that the pith was reduced by the *dig1*∆ mutant (Fig. 1G, after), which one would expect to promote the release of nutrients. Thus, one function of the fMAPK pathway is to deteriorate the pith of the rind that connects plant tissues, which may promote the release of nutrients from fruits.

### Galactose induces the fMAPK pathway in a separate way than glucose limitation or other carbon sources

Growth in the non-preferred carbon source galactose stimulates the fMAPK pathway (36, 145), which may be due to the absence of a preferred carbon source like glucose. Alternatively, galactose may specifically induce the fMAPK pathway. Due to the prevalence of galactose in yeast habitats, we tested galactose in comparison to other sugars for induction of the fMAPK pathway.

**Fig 2:**
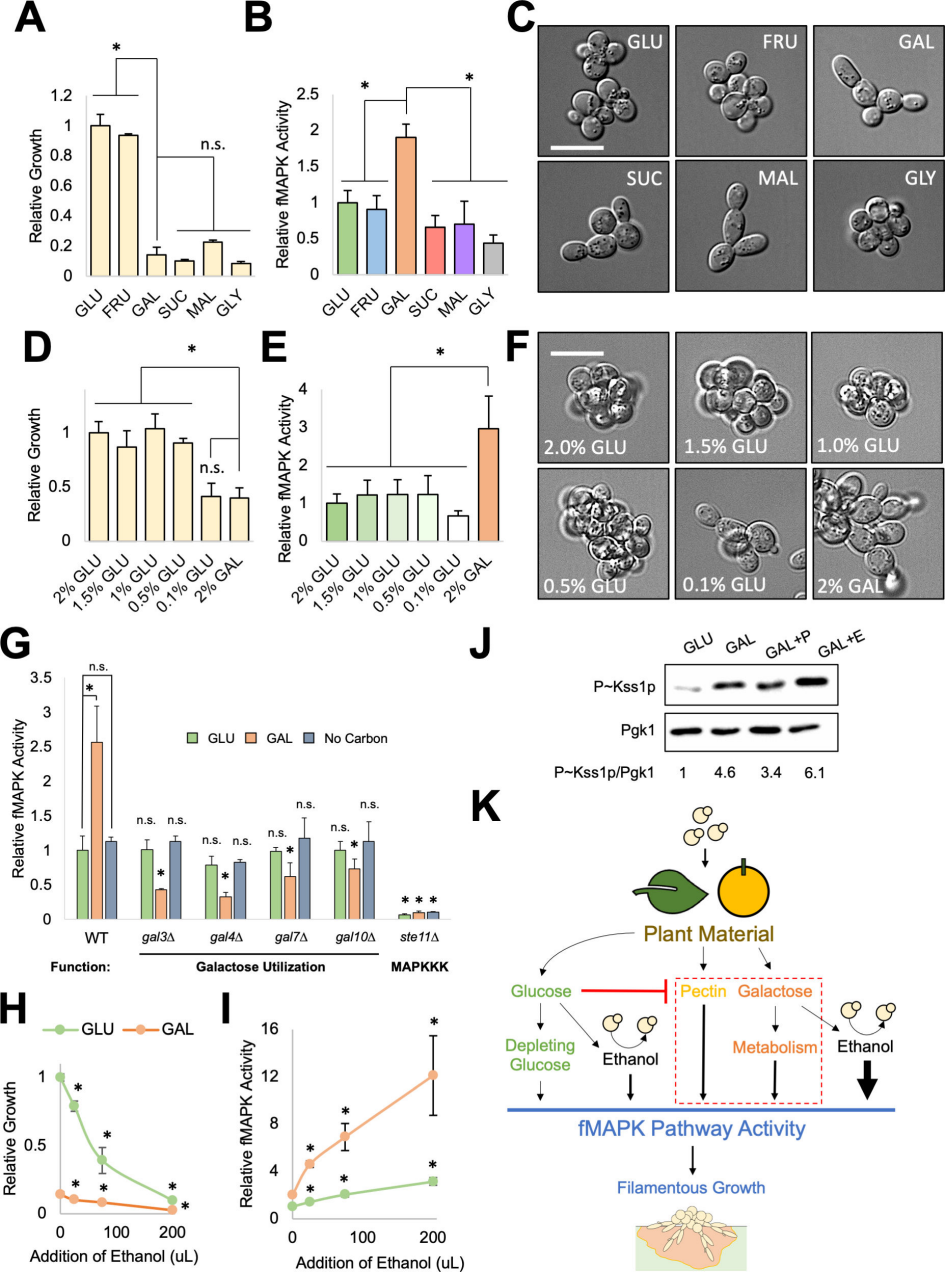
Galactose and ethanol induce the fMAPK pathway. (**A–C**) Wild-type cells (PC313) were grown in 5 mL synthetic media with indicated carbon source for 8 h. Panel A, average relative growth determined by OD_600_ of at least three replicates are reported with GLU values set to 1. Error bars represent standard deviation. *, *P*-value <0.05 by Student’s *t*-test compared to GAL. Panel B, β-galactosidase (*FRE-lacZ*) assays, average relative fMAPK pathway activity across at least three replicates is reported, with GLU values set to 1. Error bars represent standard deviation. *, *P*-value <0.05 by Student’s *t*-test to GAL. Panel C, images of cell morphology in indicated media. Bar = 10 um. (**D–F**) Wild-type cells (PC313) were grown in 2 mL synthetic media with glucose or galactose at indicated concentrations for 15 h. Panel D, average relative growth determined by OD_600_ of at least three replicates are reported with 2% GLU values set to 1. Error bars represent standard deviation. *, *P*-value <0.05 by Student’s *t*-test compared to 2% GAL. Panel E, β-galactosidase (*FRE-lacZ*) assays, average relative fMAPK pathway activity across at least three replicates is reported, with 2% GLU values set to 1. Error bars represent standard deviation. *, *P*-value <0.05 by Student’s *t*-test to 2% GAL. Panel F, images of cell morphology in indicated media. Bar = 10 um. (**G**) β-galactosidase (*FRE-lacZ*) assays. Wild-type (PC313) cells and the *ste11*∆ (PC5024), *gal3*∆ (PC7849), *gal4*∆ (PC7845), *gal7*∆ (PC7844), and *gal10*∆ (PC7846) were grown in 2 mL rich media (yeast extract, peptone) with indicated carbon source (or no carbon source as a control) for 7 h. Average relative fMAPK pathway activity across at least three replicates is reported, with wild-type values in GLU set to 1. Error bars represent standard deviation. * for wild-type values, *P*-value <0.05 by Student’s *t*-test compared to indicated condition. * for mutants’ values, *P*-value <0.05 by Student’s *t*-test compared to wild-type values from same condition. (**H–I**) Wild-type cells (PC313) were grown in glucose or galactose with the indicated concentrations of ethanol added to 5 mL cultures for 17 h. Max addition, 200 µL of ethanol, is 3.85% ethanol. Panel H, average relative growth determined by OD_600_ of at least three replicates are reported with GLU values set to 1. Error bars represent standard deviation. *, *P*-value <0.05 by Student’s *t*-test comparing tested ethanol concentration to its respective carbon source with no ethanol added. Panel I, β-galactosidase (*FRE-lacZ*) assays, average relative fMAPK pathway activity across at least three replicates is reported, with values in GLU set to 1. Error bars represent standard deviation. *, *P*-value <0.05 by Student’s *t*-test comparing tested ethanol concentration to the same carbon source with no ethanol added. (**J**) Immunoblot analysis of wild-type cells (PC313) grown in 5 mL synthetic medium with indicated carbon source for 17 h. +P = + 1% pectin. +E = +3.85% ethanol. Cell extracts were probed with antibodies to detect phosphorylated Kss1p (P ~ Kss1p) and Pgk1p as a control for protein levels. Numbers refer to the ratio of P ~ Kss1p to Pgk1p with GLU values set to 1. (**K**) Model of new inducers. Depleting glucose activates the pathway to a minor degree (smallest arrow). Pectin and galactose induce the fMAPK pathway in environments depleted for glucose. Ethanol induces the pathway in glucose and more strongly in galactose (galactose with ethanol has largest arrow). fMAPK pathway induction by these stimuli leads to filamentous growth.

Cells were examined for growth, fMAPK pathway activity, and cell morphology in preferred carbon sources where cells grew well (Fig. 2A, glucose [GLU] and fructose [FRU]), and in non-preferred carbon sources where cells grew similarly and poorly (galactose [GAL], sucrose [SUC], maltose [MAL], and glycerol [GLY]). The fMAPK pathway was induced by galactose to higher levels than by other carbon sources (Fig. 2B, 8 h). As shown previously (37), the degree of filamentous growth changed based on the carbon source tested. Cells became elongated in galactose, sucrose, and maltose, but not glucose, fructose, or glycerol (Fig. 2C). These results indicate that the morphological changes associated with filamentous growth and the activity of the fMAPK pathway can occur separately depending on the carbon source, although we show that they correlate at least for galactose (see below, % filamentous cells). This may be because different transcriptional targets or other changes at the protein level also impact morphology, which may result from the fact that other pathways also regulate filamentous growth (28, 29, 65, 95, 124 – 128, 132, 146 – 152). Glycerol failed to induce filamentous growth or the fMAPK pathway, indicating that some poor carbon sources do not trigger this differentiation response or activate the fMAPK pathway, at least at the time point tested here. At 24 h, cells grown in maltose and galactose grew similarly (Fig. S3A), and galactose and maltose both induced the fMAPK pathway relative to glucose, although galactose induced the pathway to higher levels (Fig. 1B). Therefore, galactose induced the fMAPK pathway at an earlier time point relative to other carbon sources (8 h, Fig. 2B) and to higher levels at a later time point (24 h, Fig. 1B). Thus, induction of the fMAPK pathway occurs by the addition of galactose in a manner that is distinct from other poor carbon sources.

We previously showed that glucose depletion triggers invasive growth (29). In line with this observation, fMAPK pathway activity increased over time in media containing glucose, presumably as glucose concentrations decreased (Fig. S3B). However, in addition to glucose depletion, the above findings suggest that the addition of galactose might induce the fMAPK pathway. To directly compare “glucose limitation” to “galactose induction,” different concentrations of glucose were compared for growth (Fig. 2D), fMAPK pathway activity (Fig. 2E), and cell elongation (Fig. 2F). Cells grew similarly and poorly in both 0.1% glucose and 2% galactose and showed similar cell elongation (Fig. 2D and F); however, galactose induced fMAPK pathway activity compared to glucose depletion (Fig. 2E). Surprisingly, lower glucose concentrations did not cause an increase in fMAPK pathway activity by this method (Fig. 2E, compare 2% GLU to 0.1% GLU). This may be due to the fact that when cells deplete glucose over time, there is a build-up of other signals in the environment (e.g. metabolites) that do not occur when cells are transferred to limiting glucose. Likewise, by examining fMAPK pathway activity at a time point when growth in galactose reached a comparable cell density as growth in glucose (Fig. S3A, 24 h), and nutrients have presumably been reduced naturally, galactose-dependent induction of the fMAPK pathway was higher than seen in glucose (Fig. 1B). Thus, induction of the fMAPK pathway by galactose is different and more robust than induction of the pathway by glucose limitation.

The metabolic pathway responsible for galactose uptake and utilization (153) contains proteins for galactose transport (Gal2p [154, 155]) and transduction of the galactose signal (Gal3p and Gal80p [156, 157]) to increase the activity of the transcription factor Gal4p (158, 159). Gal4p induces the expression of genes encoding enzymes required for galactose metabolism (Gal7p and Gal10p [160 – 162]). Galactose pathway mutants (*gal3*∆, *gal4*∆*, gal7*∆*,* and *gal10*∆) were required for galactose-dependent induction of the fMAPK pathway (Fig. 2G, orange) in reference to a mutant lacking a core component of the fMAPK pathway (*ste11*∆). As expected, *GAL* genes were not required for fMAPK pathway activity in medium with glucose as the carbon source or no carbon source (Fig. 2G, green and blue). These results show that galactose metabolism is required for the induction of the fMAPK pathway by galactose.

### Ethanol induces the fMAPK pathway

Ethanol is a byproduct of glycolysis and an inducer of invasive growth (40 – 42). Like galacturonic acid (Fig. 1D)*,* ethanol inhibited growth (Fig. 2H, green). However, unlike galacturonic acid (Fig. 1E), ethanol stimulated fMAPK pathway activity (Fig. 2I, green). Like pectin, ethanol-dependent induction of the fMAPK pathway was more evident in media containing galactose than glucose (Fig. 2I); however, unlike pectin, ethanol was able to induce fMAPK pathway activity in glucose to some degree (approximately threefold). In fact, induction of the fMAPK pathway by ethanol in glucose occurred to comparable levels as induction by galactose (Fig. 2I, compare GLU with ethanol to GAL without ethanol). Thus, ethanol induces the fMAPK pathway (and causes increased filamentous growth in some contexts, see below, % filamentous cells).

Phosphorylation of the MAP kinase Kss1p (P ~ Kss1p) is induced by activation of the fMAPK pathway and provides an additional readout of fMAPK pathway activity. As previously shown (36, 64, 145), galactose caused an increase in P ~ Kss1p levels relative to glucose (Fig. 2J). Ethanol further increased P ~ Kss1p levels relative to galactose (Fig. 2J). Pectin did not induce P ~ Kss1p levels relative to galactose by this assay, which may be because pectin induces the pathway at different time points than those tested here. Pectin, galactose, and ethanol induced the fMAPK pathway by a second transcriptional reporter (*NFG1-lacZ* [163] [Fig. S4A]), which is a strongly induced target of the fMAPK pathway (48, 145, 163, 164).

Yeast has a haploid and diploid stage in their life cycle (165 – 167) and likely prefers the diploid state because they are typically diploid when isolated from the wild (108, 112, 119, 168) and mate readily if able (169, 170); therefore, we tested for fMAPK pathway induction in diploid cells and found that pectin, galactose, and ethanol also activate the fMAPK pathway in diploids (Fig. S5). Collectively, the results presented here show that the fMAPK pathway can be induced by stimuli (pectin, galactose, and ethanol) that might be commonly encountered when cells are exposed to and metabolize plant material in natural settings. Taking into account the dependency on glucose limitation for several of the inducers, we suggest a model for how these stimuli might be encountered (Fig. 2K).

### Combinations of inducers stimulate the fMAPK pathway to near-maximal levels and reveal environment-dependent roles for pathway components

**Fig 3:**
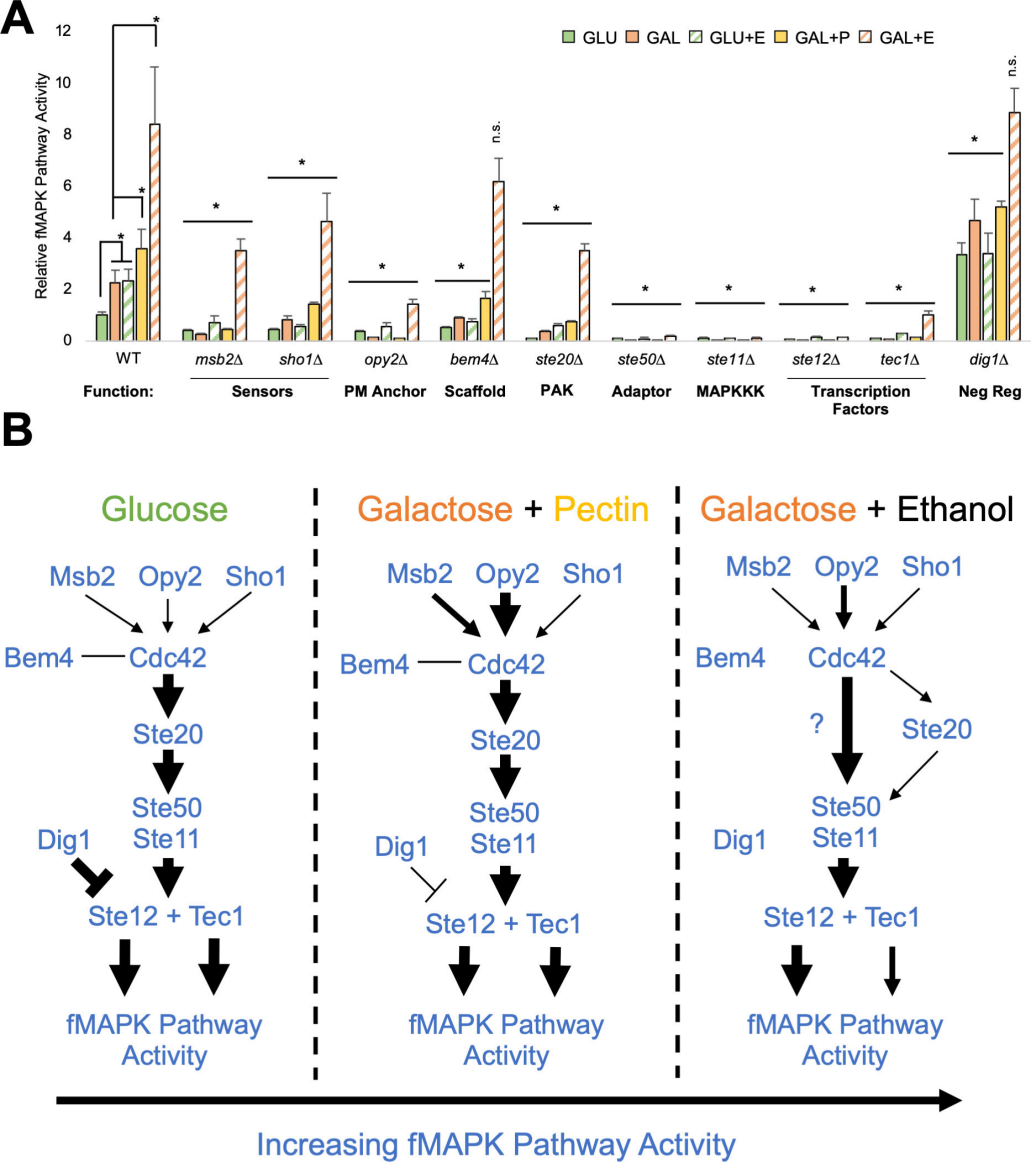
Combinations of inducers activate the fMAPK pathway to near-maximal levels and reveal roles of fMAPK pathway components under different conditions. (**A**) β-galactosidase (*FRE-lacZ*) assays. Wild-type cells (PC313) and the *msb2*∆ (PC961), *opy2*∆ (PC3894), *sho1*∆ (PC5026), *bem4*∆ (PC3343), *ste20*∆ (PC7871), *ste50*∆ (PC4982), *ste11*∆ (PC5024), *ste12*∆ (PC5651), *tec1*∆ (PC7675), and *dig1*∆ (PC7676) mutants were grown in 2 mL synthetic media with indicated carbon source for 17 h. +P = + 1% pectin. +E = +3.85% ethanol. Average relative fMAPK pathway activity across at least three replicates is reported, with wild-type values in GLU set to 1. Error bars represent standard deviation. * for wild-type values, *P*-value <0.05 by Student’s *t*-test compared to indicated condition. * for mutants’ values, *P*-value <0.05 by Student’s *t*-test compared to wild-type values from same condition. (**B**) Model depicting findings in panel A. Arrow size represents MAPK activity based on the data in panel 3A.

Multiple inducers might be encountered in the wild and were therefore tested in combinations. As shown above in galactose, pectin or ethanol stimulated fMAPK pathway activity above galactose alone (Fig. 3A, WT). These results suggest that pectin and ethanol have additive effects with galactose. When galactose, pectin, and ethanol were combined, there was no further increase in fMAPK pathway activity above galactose with ethanol (Fig. S6), suggesting pectin does not necessarily have an additive effect with ethanol. Alternatively, the addition of pectin to galactose with ethanol may not further stimulate pathway activity because the pathway may be maximally activated. To test this hypothesis, wild-type cells were compared to the *dig1*∆ mutant, which shows very high fMAPK pathway activity seen in laboratory settings (124). The *dig1*∆ mutant showed elevated fMAPK pathway activity compared to wild-type cells in most conditions, except in galactose with ethanol (Fig. 3A, compare wild type to the *dig1*∆ mutant, galactose with ethanol, striped orange) or when all three inducers were combined (Fig. S6). These results show that the fMAPK pathway can be activated to near-maximal levels when cells encounter a combination of inducers (Fig. 2J, galactose with ethanol, largest-inducing arrow).

We next tested how different inducers may be sensed and relayed by fMAPK pathway components (Fig. 1A). In general, cells lacking fMAPK pathway regulatory proteins (*msb2*∆*, opy2*∆*, sho1*∆*, bem4*∆*, ste20*∆*, ste50*∆*, ste11*∆*, ste12*∆*,* and *tec1*∆ mutants; *cdc24*∆ and *cdc42*∆ mutants are inviable and were not tested) showed reduced fMAPK pathway activity compared to wild type in response to the inducers tested (Fig. 3A). However, the proteins that reside at the plasma membrane, Msb2p, Sho1p, and Opy2p were more critical for signaling in some environments than others. For example, the *msb2*∆ mutant showed a stronger reduction in fMAPK pathway activity in galactose compared to galactose with ethanol relative to wild-type levels (Fig. 3A, galactose, *msb2*∆ shows ~10-fold reduction compared to WT, galactose with ethanol, *msb2*∆ shows ~2.5-fold reduction compared to WT).

Msb2p, Sho1p, and Opy2p also showed different requirements for signaling compared to each other depending on the environment. For example, the *msb2*∆, *sho1*∆, and *opy2*∆ mutants had a similar reduction in fMAPK pathway activity in glucose (Fig. 3A and B); however, the *opy2*∆ mutant showed a stronger reduction than the *msb2*∆ or *sho1*∆ mutants in other environments (Fig. 3A and B, *P*-values by Student’s *t*-test, galactose with pectin, *P*-value <0.0002 to *msb2*∆ and <0.00001 to *sho1*∆, galactose with ethanol, *P*-value <0.003 to *msb2*∆ and <0.008 to *sho1*∆). The observation that Opy2p showed a stronger role than Msb2p or Sho1p is in agreement with previous results (36), tying Opy2p to Ste11p recruitment and activation (59, 75, 171, 172). We also noticed that in response to galactose with pectin, the *msb2*∆ mutant showed a larger reduction in fMAPK pathway activity than the *sho1*∆ mutant (Fig. 3A and B, galactose with pectin, *P*-value <0.00001 by Student’s *t*-test), whereas the *msb2*∆ and *sho1*∆ mutants showed similar reduction in galactose with ethanol. Overall, these findings suggest that the stimuli tested may be sensed in different ways by different proteins.

Similarly, some proteins were required for signaling in all conditions tested (Fig. 3A, Ste50p, Ste11p, and Ste12p); whereas others were partially or completely dispensable in certain contexts. For example, Msb2p, Sho1p, and Opy2p were partly dispensable in most environments, especially under maximally inducing conditions (Fig. 3A and B, galactose with ethanol). In galactose with ethanol, Ste20p was also partially dispensable (Fig. 3A and B) and the Bem4p and Dig1p proteins were fully dispensable (Fig. 3A and B). We also found that the two main transcription factors for the fMAPK pathway, Tec1p and Ste12p, which normally have the same phenotype, showed different requirements for fMAPK pathway activity. Ste12p was absolutely required for signaling in galactose with ethanol, but Tec1p was not (Fig. 3A and B, *P*-value <0.0004 by Student’s *t*-test comparing *tec1*∆ and *ste12*∆). This was also seen in the diploid strain (Fig. S5). Collectively, these data show that the requirement for a subset of fMAPK pathway components changes depending on the condition tested.

### Components of the fMAPK pathway show variation in regulating filamentous growth

The fMAPK pathway regulates differentiation to the filamentous cell type (28, 29). Cell differentiation includes an elongation of cell shape, due to a delay in the G1 and G2 phases of the cell cycle (48, 173, 174), and in haploid cells, a switch from axial budding (growth toward the mother cell) to distal budding [or growth away from the mother cell (28, 35, 175, 176)]. These features were observed in response to combinations of inducers by microscopy and represented as a ratio of cells undergoing filamentous growth compared to the total number of cells (% filamentous). A cell was considered filamentous if it exhibited an elongated cell morphology or distal budding pattern (see Material and Methods).

**Fig 4:**
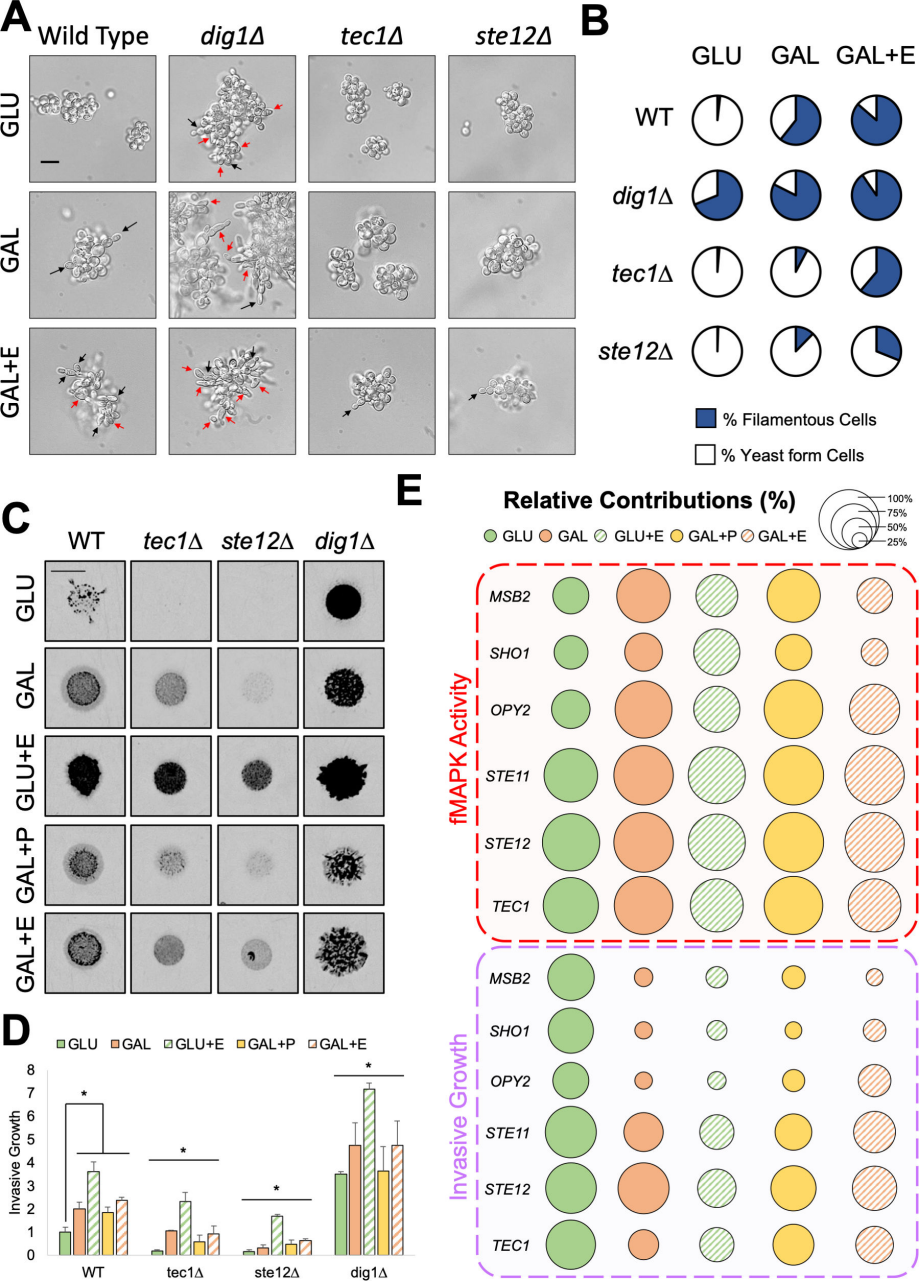
Environment-dependent roles for pathway components in filamentous growth. (**A**) Wild-type (PC313) cells and the *ste12*∆ (PC5651), *tec1*∆ (PC7675), and *dig1*∆ (PC7676) mutants were examined by microscopy in indicated media and imaged. Microscopy images taken at 100× in indicated media. Bar, 10 um. Black arrow, filamentous cell. Red arrow, cell displaying aberrant morphology. (**B**) Quantified % filamentous cells. Representative images in panel A. % filamentous cells (blue). % yeast-form cells (white). (**C**) Plate-washing assay. Images, wild-type cells (PC313) and the *ste12*∆ (PC5651), *tec1*∆ (PC7675), and *dig1*∆ (PC7676) mutants were spotted for 5 d on indicated medium. Inverted images of invasive scars are shown. Bar, 0.5 cm. Before wash images of colonies are shown in Fig. S8A. Results slightly differ from those published in reference 124 because they were performed for 5 d here instead of 7 d in the previous study. (**D**) Quantitation of invasive growth in panel C. Average relative invasion across at least three replicates is reported, with wild-type values in GLU set to 1. Error bars represent standard deviation. Asterisk for wild-type values, *P*-value <0.05 by Student’s *t*-test compared to indicated condition. Asterisk for mutants’ values, *P*-value <0.05 by Student’s *t*-test compared to wild-type values from same condition. (**E**) Relative % contribution for fMAPK pathway activity (top) or invasive growth (bottom) in the indicated conditions, where circle size represents the level of contribution. Larger circle, larger contribution. Contribution is determined as reported (124) based on the reduction in invasive growth or fMAPK pathway activity relative to wild type converted to a percentage. Based on quantitation in Fig. S8B and C.

For wild-type cells, the % filamentous cells increased from growth in glucose to growth in galactose media and from galactose to galactose with ethanol media (Fig. 4A, representative images, additional images in Fig. S7; Fig. 4B, quantitation). This increase correlated to fMAPK pathway activity (Fig. 3A). The length of individual cells did not increase from galactose to galactose with ethanol (Fig. 4A, WT, black arrows, compare GAL to GAL + E) even though fMAPK pathway activity increased between these environments (Fig. 3A). These data suggest that the increase in fMAPK pathway activity may result from a higher number of cells in the population being stimulated, rather than a higher level of fMAPK pathway activity in individual cells. Moreover, the *dig1*∆ mutant was similar to wild-type cells in the maximally inducing condition (galactose with ethanol) for both fMAPK pathway activity (Fig. 3A) and the % filamentous cells (Fig. 4B). Notably, the *dig1*∆ mutant did show more aberrant cell morphologies than wild-type cells (Fig. 4A, compare red arrows), which may reflect that cells can tolerate fMAPK pathway activation by a natural stimulus compared to hyperactivation resulting from a genetic perturbation. This is possibly because Dig1p may have pleiotropic effects on cell morphology unrelated to its role in regulating fMAPK pathway activity.

For cells lacking Ste12p and Tec1p in galactose with ethanol, the % filamentous cells (Fig. 4B) matched fMAPK pathway activity (Fig. 3A), in that the *ste12*∆ mutant showed less % filamentous cells than the *tec1*∆ mutant. In this context, the *tec1*∆ and *ste12*∆ mutants showed some cell elongation and distal budding (Fig. 4A, GAL + E, black arrows), which is likely due to another pathway that regulates filamentous growth. Thus, we identified a condition where Tec1p can be partly by-passed for both fMAPK pathway activity (Fig. 3) and filamentous growth (Fig. 4A and B).

Filamentous growth can also be visualized by the plate-washing assay, where spotted cells washed with a stream of water show invasive growth (45). Invasive growth is normally tested on YPD (yeast extract, peptone, dextrose) media, but here, synthetic medium was used to match conditions where the *FRE* reporter was evaluated. The plate-washing assay showed that components of the fMAPK pathway were required for invasive growth more under some conditions than others (Fig. S8A and B). For example, the plate-washing assay supported the finding that Ste12p plays a more critical role than Tec1p under several conditions (Fig. 4C, e.g., GAL, quantitation in Fig. 4D), which supports fMAPK pathway activity (Fig. 3A) and % filamentous cells (Fig. 4B) data above.

Invasive growth matched or showed differences compared to fMAPK pathway activity depending on the environment (direct comparison between fMAPK activity and invasion in Fig. S8B through D). For example, invasive growth (Fig. 4C and D, WT, compare down columns) matched fMAPK pathway activity (Fig. 3A) based on the fact that galactose stimulated both phenotypes relative to glucose, and glucose with ethanol stimulated both phenotypes relative to glucose alone. Invasive growth differed from fMAPK pathway activity based on the fact that glucose with ethanol induced the most invasion (Fig. 4C and D, WT), whereas galactose with ethanol induced the most fMAPK pathway activity (Fig. 3A, WT). In addition, combinations of galactose plus pectin or ethanol did not cause an increase in invasive growth beyond galactose alone (Fig. 4C and D, WT) as it did for fMAPK pathway activity (Fig. 3A, WT). These results suggest that fMAPK pathway activity and invasive growth are separable phenotypes. This idea was not noted in our previous study (124), most likely because environments were not examined that maximally activate the fMAPK pathway. This result may suggest that fMAPK pathway activity and invasive growth become uncoupled at higher levels of pathway activation.

Moreover, some fMAPK pathway components made different contributions toward those two phenotypes depending on the environment (Fig. 4E, plate-washing assay images and quantification in Fig. S8). For example, Msb2p, Sho1p, and Opy2p generally showed similar contributions to both the regulation of fMAPK pathway activity and invasion in glucose (Fig. 4E, GLU, green, circle sizes are similar between fMAPK pathway activity (top) and invasive growth [bottom]) but showed differences in their contributions between the two phenotypes in inducing environments (Fig. 4E, e.g., GAL, orange, circle sizes are much larger for fMAPK activity [top] than invasive growth [bottom]). The downstream components (Ste11p, Ste12p, and Tec1p) were also variable in their contribution between the two phenotypes, but to a lesser degree (Fig. 4E). Invasive growth may be less affected by fMAPK pathway perturbation than fMAPK pathway activity in some contexts because invasive growth is regulated by other pathways (27, 177). Overall, the findings in this section suggest that environmental inducers stimulate invasive growth, cell differentiation, and fMAPK pathway activity, but not necessarily in the same manner, because fMAPK pathway components show variation between each other in some contexts.

### A key role for the Ras2-PKA pathway in the conditional regulation of the fMAPK pathway

**Fig 5:**
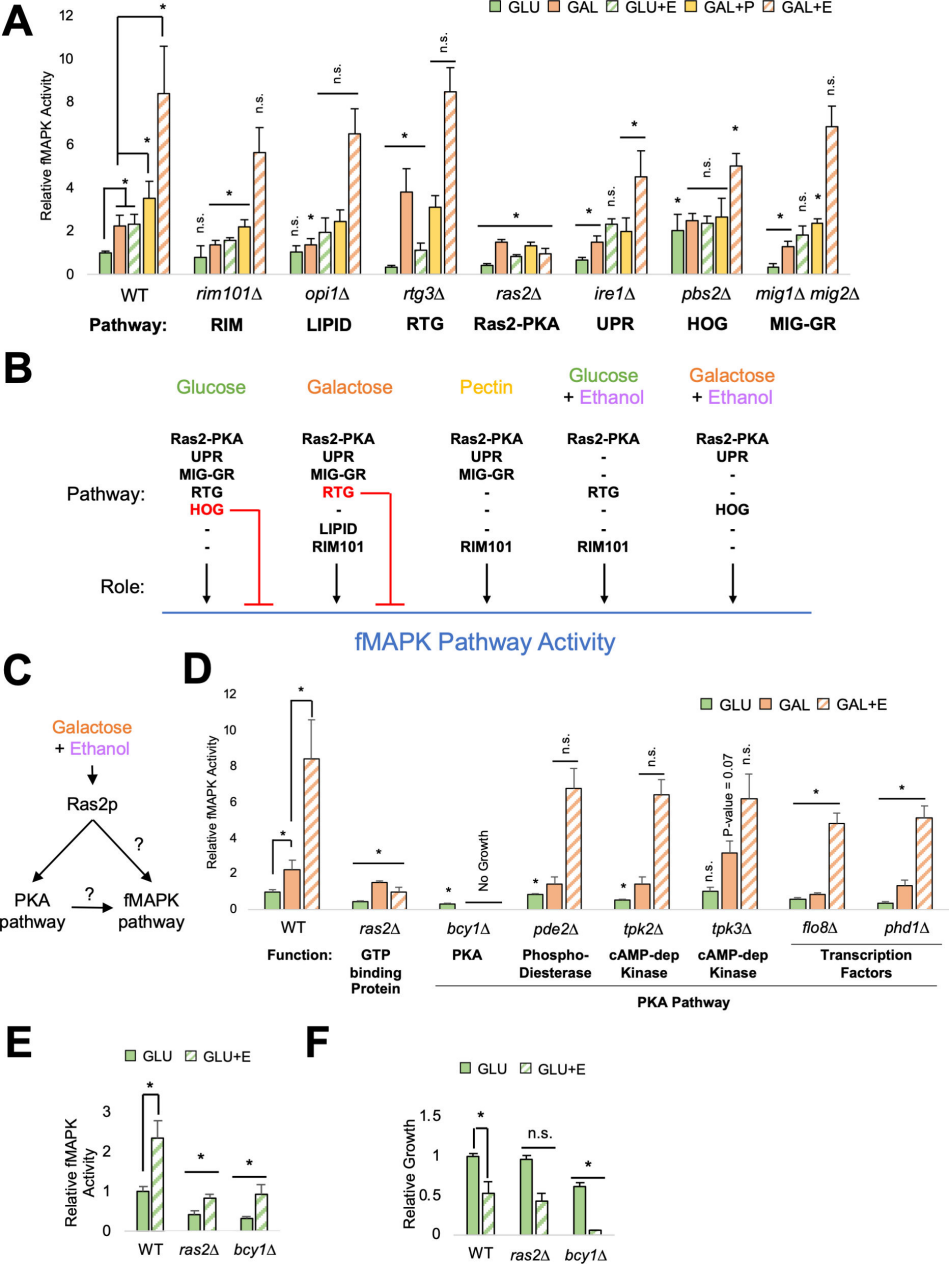
Role for the Ras2-PKA pathway in the conditional regulation of the fMAPK pathway. (**A**) β-galactosidase (*FRE-lacZ*) assays of mutants lacking pathways that regulate filamentous growth. Wild-type (PC313) cells and the *rim101*∆ (PC7673), *opi1*∆ (PC7674), *rtg3*∆ (PC7677), *ras2*∆ (PC6222), *ire1*∆ (PC6048), *pbs2*∆ (PC5035), and *mig1*∆ *mig2*∆ (PC5187) mutants were grown in 2 mL synthetic media with indicated carbon source after 17 h. +P = + 1% pectin. +E = +3.85% ethanol. Average relative fMAPK pathway activity across at least three replicates is reported, with wild-type values in GLU set to 1. Error bars represent standard deviation. * for wild-type values, *P*-value <0.05 by Student’s *t*-test compared to indicated condition. Asterisk for mutants’ values, *P*-value <0.05 by Student’s *t*-test compared to wild-type values from same condition. Wild-type values are the same as shown in Fig. 3A. (**B**) Model of network-level regulation of fMAPK pathway activity. Black arrow, positive role. Red arrow, inhibitory role. Dash line, pathway does not serve a role in indicated condition. (**C**) Model of possible Ras2-PKA pathway regulation of the fMAPK pathway in galactose with ethanol. (**D**) β-galactosidase (*FRE-lacZ*) assays of the PKA pathway. Performed same as panel A except wild-type cells (PC313) and the *ras2*∆ (PC6222), *bcy1*∆ (PC7870), *pde2*∆ (PC7872), *tpk2*∆ (PC7874), *tpk3*∆ (PC7869), *flo8*∆ (PC7865), and *phd1*∆ (PC7873) mutants were used. The *ras2*∆ mutant values are the same as shown in panel A. (**E**) β-galactosidase (*FRE-lacZ*) assays. Same as panel A except wild-type (PC313) cells and the *ras2*∆ (PC6222) and *bcy1*∆ (PC7870) mutants were used. The *ras2*∆ mutant values are the same as shown in panel A. The *bcy1*∆ mutant value in GLU is the same as shown in panel C. (**F**) Relative growth of wild-type (PC313) cells and the *ras2*∆ (PC6222) and *bcy1*∆ (PC7870) mutants in 2 mL synthetic media with indicated carbon source after 17 h. Average relative growth across at least three replicates is reported, with wild-type GLU values set to 1. Error bars represent standard deviation. * for wild-type values, *P*-value <0.05 by Student’s *t*-test compared to indicated condition. * for mutants’ values, *P*-value <0.05 by Student’s *t*-test compared to wild-type values from same condition.

Other pathways regulate filamentous growth including a major nutrient-sensing pathway called the Ras2p cAMP-dependent PKA pathway (Ras2-PKA [28, 124 – 128]), the RIM101 pathway [RIM] [146, 147], the retrograde pathway [RTG] [124, 148, 149], the unfolded protein response [UPR] [65], the phospholipid biosynthesis regulator, Opi1p [LIPID] [124, 150], the high-osmolarity glycerol response pathway [HOG] [95, 151, 152], and the Snf1p or AMP-dependent kinase [AMPK] and the Mig1/2p-GR pathway [MIG-GR] [29, 132]). Furthermore, the abovementioned pathways also regulate the fMAPK pathway (124, 125, 178). Key mutants known to ablate the Ras2-PKA, RIM, RTG, UPR, LIPID, HOG, and MIG-GR pathways were examined for fMAPK pathway activity under the conditions examined in this study. Most pathways played distinct roles in regulating the fMAPK pathway depending on the condition tested. For example, the Ras2-PKA pathway was required under five conditions, whereas the UPR was only required under four conditions, and the RIM pathway was only required under three conditions (Fig. 5A and B). Moreover, some pathways played a positive role in regulating the fMAPK pathway in one environment and a negative role in another (Fig. 5A and B, e.g., RTG: positive, GLU, negative, GAL; HOG: positive, GAL + E, negative, GLU). A “role reversal” has previously been reported for the RTG pathway in multiple environments for invasive growth (124). Strikingly, the Ras2-PKA pathway played a central role in regulating fMAPK pathway activity in galactose with ethanol, even more than Msb2p, Sho1p, Bem4p, or Ste20p (Ras2-PKA in Fig. 5A, fMAPK components in Fig. 3A). The Ras2-PKA pathway also played a major role in invasive growth and showed a stronger contribution in response to ethanol (but not the absence of ethanol) than any component of the fMAPK pathway tested (Fig. S8, *ras2*∆). This result suggests that the Ras2-PKA pathway is required for fMAPK pathway activity in contexts with ethanol, rather than functioning as an ancillary pathway that augments core pathway activity.

The Ras2-PKA pathway may regulate the fMAPK pathway in environments with ethanol by direct interactions between fMAPK pathway components and the GTPase Ras2p (128, 129) or through Ras2p regulation of the PKA pathway (Fig. 5C) (126, 127). Strains lacking PKA components including the cAMP-dependent kinases, Tpk2p and Tpk3p (128, 179, 180), the transcription factors, Flo8p and Phd1p (60, 99, 181 – 183), the phosphodiesterase, Pde2p (34, 184 – 186), and the regulatory subunit of PKA, Bcy1p (181, 187), were generated and tested for fMAPK pathway activity in response to galactose with ethanol (Fig. 5D). Some PKA mutants showed a reduction (*tpk2*∆ and *pde2*∆, GLU) or increase (*tpk3*∆, GAL) in fMAPK pathway activity in one environment (Fig. 5D). Others showed a decrease across all environments tested (*flo8*∆*, phd1*∆*,* Fig. 5D). The deletion of the gene encoding Bcy1p, the regulatory subunit of PKA (181, 187), showed a strong and comparable reduction as the *ras2*∆ mutant for fMAPK pathway activity in glucose (Fig. 5E, green), though it also showed reduced growth (Fig. 5F, green). The *bcy1*∆ mutant did not grow in galactose, so we tested the mutant in glucose with ethanol, where it showed a similar result (Fig. 5E and F). Thus, both Ras2p and PKA pathway disruptions reduced fMAPK pathway activity in response to ethanol. Two-hybrid tests did not identify an interaction between Ras2p and fMAPK pathway components (Fig. S9); however, Ras2p may directly regulate the fMAPK pathway as it interacts with Cdc24p (188) and the polarity adaptor Bem1p (83 – 85) which both regulate the fMAPK pathway (83, 86). Thus, the Ras2-PKA pathway is important for fMAPK pathway activity in response to ethanol, including conditions where the pathway functions near-maximal levels (e.g., galactose with ethanol).

## DISCUSSION

*S. cerevisiae*, like pathogens including *C. albicans* and *U. maydis*, undergoes a type of filamentous growth that is regulated by a MAPK pathway when exposed to specific environments. Unlike pathogens which are commonly studied in their hosts, MAPK pathway function in *S. cerevisiae* has been studied mostly under standard laboratory conditions. To examine the fMAPK pathway from an ecological perspective, we tested compounds that may be encountered by *S. cerevisiae* in the wild (e.g., plant-derived compounds). New inducers of the fMAPK pathway were identified (pectin, galactose, and ethanol, Fig. 2K), that in certain combinations stimulated the pathway to high levels. By examining this “maximal” MAPK signaling, conditional roles for pathway regulators were identified. Thus, an ecological perspective led to new insights about the induction and regulation of a signal transduction pathway.

We found that pectin induces the fMAPK pathway. This connection suggests a role for the pathway in plant-environment recognition. Because the fMAPK pathway controls expression of the cell’s major pectinase (48), detecting pectin may lead to the breakdown of plant material to release sugars for scavenging. We showed that yeast is effective at the degradation of the pith of fruits, which may promote the release of nutrients. Of the sugars expected to be released, glucose is the preferred carbon source in yeast and prevents the use of non-preferred carbon sources (e.g., galactose, sucrose, maltose [132, 189, 190]). We found that glucose also prevents pectin induction of the fMAPK pathway, which may be because filamentous growth evolved to occur in the absence of a preferred carbon source. Additionally, we found that galacturonic acid, a compound that may be introduced into the environment when other microbes forage and break down pectin (136 – 139), inhibited growth and fMAPK pathway activity.

We also found that galactose is more effective at activating the fMAPK pathway than glucose limitation or other non-preferred carbon sources. The differences between effects on fMAPK pathway activity by glucose, maltose, galactose, and sucrose may be because the sugars are metabolized differently, require different enzymes and permeases, and enter glycolysis in different ways (30). Because galactose is found in abundance in plant material (e.g., fruits and forest floors), this sugar may signal that cells have encountered an environment with a suitable carbon source for scavenging even in the absence of a preferred carbon source. Similarly, galactose and pectin induce the secretion of pectolytic enzymes in distantly related filamentous fungi, like *Neurospora crassa*, where glucose also inhibits pectin induction (191 – 194).

One byproduct of carbon source utilization in *S. cerevisiae* is ethanol, which accumulates over time and can indicate the decline in available nutrients. Ethanol also acts as a quorum-sensing molecule (43). Ethanol has previously been shown to induce filamentous growth (41), and we show that ethanol also stimulates the fMAPK pathway. The fact that ethanol induces fMAPK pathway activity in glucose suggests that it is an independent signal from nutrient levels and likely a signal about increasing cell density. In addition, ethanol induced the fMAPK pathway to higher levels in galactose, which suggests that it may also act to amplify the low-nutrient signal. Thus, ethanol may integrate two important signals, low nutrients and high cell density.

It is not yet clear how pectin, galactose, and ethanol are “sensed” by the fMAPK pathway. Pectin may be sensed by Msb2p at the cell surface, as some mammalian mucins expressed in gut tissue interact with pectin (195, 196). Large pectin molecules may act as a scaffold by interacting with and bringing signaling complexes together (e.g., Msb2p, Sho1p, and Opy2p). How pectin activates Msb2p and Sho1p is of interest because Msb2p- and Sho1p-like proteins also regulate MAPK pathways involved in host recognition and invasion in plant pathogens like *U. maydis* (24, 25), *Fusarium oxysporum* (22), *Magnaportha oryzae* (23), and *Colletotrichum gloeosporioides* (21). We also showed that galactose signaling requires galactose metabolism. This may suggest that one of the components of the galactose utilization pathway may interact with the cytosolic domains of Msb2p and/or Sho1p or that a small metabolite is involved. Ethanol may also be detected by sensors through its ability to disrupt the structure of cell membranes, which is where the fMAPK pathway sensors are located, or its ability to denature proteins (197, 198).

Previously, we showed that most conditions induced the fMAPK pathway to less than half-maximal levels (124). Here, the ecologically relevant stimuli in combination were able to maximally activate the fMAPK pathway. This did not cause defects in cell morphology and budding as seen in mutants that hyperactivate the fMAPK pathway (64, 178, 199). This maximally activated state also revealed that fMAPK pathway activity in certain contexts is separable from some phenotypes (e.g., invasive growth). Broadly speaking, these findings could suggest two things relative to other MAPK pathways and organisms. First, this could imply that MAPK pathway activity and output phenotypes do not directly correlate as one might predict at different levels of activation. Second, the prior techniques of studying MAPK states through the introduction of hyperactive alleles of pathway components or the deletion of negative regulators may not fully represent what cells do when a MAPK pathway is activated. Therefore, the ability to activate the fMAPK pathway to high levels with commonly encountered stimuli without generating obvious morphological abnormalities represents a new and powerful tool for molecular dissection of pathway function.

This ability to activate the fMAPK pathway to high levels uncovered conditional differences for fMAPK pathway components. These included conditional differences for the sensors Msb2p, Sho1p, and Opy2p, the scaffold Bem4p, the negative regulator Dig1p, and the PAK kinase Ste20p (Fig. 3B). Ste20p has previously been found to be dispensable for ethanol-dependent filamentous growth compared to other pathway components (41), can be bypassed by activated versions of Cdc42p (200), and is partly redundant in a related signaling pathway that responds to osmotic stress (71). One explanation for the conditional roles for these proteins is by the action of other pathways. For example, the Ras2-PKA pathway played a more critical role in regulating fMAPK pathway activity in the maximally inducing environment compared to canonical fMAPK pathway components (Msb2p, Sho1p, Ste20p, and Bem4p), suggesting that Ras2-PKA may bypass these proteins in a condition-specific manner.

The transcription factors Ste12p and Tec1p work mostly in concert during filamentous growth due to the formation of a heterodimer to regulate gene expression (201). However, the proteins can also act independent of each other. Tec1p can regulate filamentous growth independently of Ste12p (98, 202, 203). Here, we show that Ste12p can play a more central role than Tec1p under some conditions. Thus, Ste12p may regulate select filamentation target genes independently of Tec1p. Tec1p and Ste12p differences may be due to their regulation by unique mechanisms. For example, Ste12p associates with the transcription factor Mcm1p (201) and is phosphorylated by the cyclin-dependent kinase Cdk8p (204). Furthermore, Tec1p is regulated by SUMOylation to modulate its role in invasive growth (205).

In conclusion, studying *S. cerevisiae* from an ecological perspective has led to new insights about signaling pathway induction and regulation. These findings may apply generally to other systems, including animal and plant pathogens who interact with hosts in unique environments through evolutionarily conserved signaling pathways (e.g., *C. albicans* [206 – 208]). For example, our findings suggest that targeting core components of a pathway may be more effective at ablating signaling than targeting sensor proteins. Broadly speaking, studying signaling pathways in diverse and relevant environments may reveal environment-dependent roles for pathway components that broaden our understanding of health, disease, and evolution.

## MATERIALS AND METHODS

### Yeast strains and plasmids

Yeast strains are listed in Table S1. Experiments were performed in haploid cells unless otherwise noted. The p*FRE-lacZ* plasmid (131) (provided by H. Madhani [UCSF]) or the p*NFG1-lacZ* plasmid (163) (provided by C. Boone) is used to measure the transcriptional activity of the fMAPK pathway. Gene deletions in haploid cells were made in the Σ1278b strain background (28) through homologous recombination, constructed using an antibiotic resistance marker (neurotactin or gentamicin) amplified by polymerase chain reaction (PCR) and introduced into yeast by lithium acetate transformation as described (209). Gene deletions in diploids were made using the CRISPR-Cas9 system (210) as has been previously described with the p*Cas* plasmid (211). For CRISPR-Cas9, the sgRNA sequence was designed using CRISPRdirect (https://crispr.dbcls.jp/) and cloned into the p*Cas* plasmid by PCR as previously described (211). To generate the p*Cas9*-sg*TEC1* plasmid, the following primers were used: forward, TTCGTATTCACAGTCGGCCTGTTTTAGAGCTAGAAATAGC, and reverse, AGGCCGACTGTGAATACGAAAAAGTCCATTCGCCACCCG. To generate the p*Cas9*-sg*STE12* plasmid, the following primers were used: forward, CCTATGATAACGTGAATGAAGTTTTAGAGCTAGAAATAGC, and reverse, TTCATTCACGTTATCATAGGAAAGTCCCATTCGCCACCCG. The sgRNA sequence was verified by sequencing the plasmid with GENEWIZ (https://www.genewiz.com/) using the sequencing primer CGGAATAGGAACTTCAAAGCG (211). The markerless homologous directed recombination template for generating deletion mutants was designed as previously described (211) as a 140-mer by using three 60-mer oligonucleotides amplified by PCR. All gene deletions were verified by PCR amplification and gel electrophoresis of deletion site and by phenotype when possible. PCR primers for homologous recombination can be found in Table S2.

### Media

Synthetic complete medium (GLU, FRU, GAL, SUC, MAL, GLY) included 0.67% yeast nitrogen base without amino acids, amino acids, and one of the following: 2% dextrose, 2% fructose, 2% galactose, 2% sucrose, 2% maltose, or 2% glycerol, respectively (minus uracil when selecting for p*FRE-lacZ* plasmid or minus leucine when selecting for p*NFG1-lacZ*). For some experiments with GLU medium, the dextrose concentration varies as indicated. For media with pectin (+P), a 1% solution was made using citrus pectin from Spectrum catalog #PE100 (https://www.spectrumchemical.com/pectin-citrus-usp-pe100); YPD was 1% yeast extract, 2% peptone, 2% dextrose; YPGAL was YPD with 2% galactose instead of dextrose. When solid media were made, 2% agar was used.

### Measurement of fMAPK pathway activity

The fMAPK pathway activity was analyzed by the β-galactosidase (*lacZ*) assay as previously described (212, 213) using a transcriptional reporter (p*FRE-lacZ* or *pNFG1-lacZ*) as the readout of fMAPK pathway activity. Experiments were done with the *FRE-lacZ* reporter except where indicated. Cells were grown in indicated medium (without uracil or leucine when maintaining selection for plasmids). Cells were grown to time points indicated and harvested by centrifugation and stored at −80^o^ for at least 30 min. Subtle variation in fMAPK pathway induction or growth by inducers can be noted across some figures. This is due to differences between experiments, as indicated in figure legends, related to different times cells were grown or different volumes of medium. The β-galactosidase (*lacZ*) assay was performed with at least three biological replicates where the average is reported and error bars represent standard deviation. Differences in values from a previous study (124) for wild type and the *ras2*∆, *rim101*∆, and *opi1*∆ mutants may be due to differences in starting cell density and incubation times.

The fMAPK pathway activity was also analyzed by phosphoblot as previously described (164) by established protocols (145, 214). Phosphorylated Kss1p was detected by p42/p44 antibodies (#4370; Cell Signaling Technology, Danvers, MA) as the primary antibody and goat anti-rabbit IgG-HRP (#111-035-144; Jackson ImmunoResearch Laboratories, West Grove, PA) as the secondary antibody. The loading control, Pgk1p, was detected by mouse α-Pgk1p antibodies (#459250; Thermo Fisher Scientific, Rockford, IL) as the primary antibody and goat α-mouse (#170-6516; Bio-Rad Laboratories) as the secondary antibody. The blot was imaged by a ChemiDoc XRS+ molecular imager and signal intensity was measured by using the volume tool in the program Image Lab (https://www.bio-rad.com/en-us/product/image-lab-software?ID=KRE6P5E8Z).

### Quantification of phenotypes

Images of cells were taken at 100× magnification by microscopy using differential interference contrast imaging with a Zeiss Axioplan 2 microscope. Digital images were acquired with the Axiocam MRm camera. Some photos were taken by an iPhone 13 (Apple) through the microscope lens. For image analysis, Axiovision 4.4 software was used.

For cell differentiation quantification, cells were observed at 100× magnification. At least 85 cells were examined for each strain. Cells that showed elongation and/or cells that budded distally were considered filamentous, whereas cells that were round and budding axially (back toward mother cell) were considered yeast form. The filamentous cells and yeast form cells were expressed as a percentage of total cells.

To measure invasive growth, cells were spotted onto indicated medium and grown for 5 d. The plate-washing assay was performed as described (35, 215). Invasive growth was quantified as described using Image Lab (https://www.bio-rad.com/en-us/product/image-lab-software?ID=KRE6P5E8Z) to calculate (volume/area)/ 10,000 (124). Invasion values were averaged across at least three biological replicates and error was determined by standard deviation.

Pectin breakdown was determined by viscosity using a drop assay after cells were grown in 10 mL of indicated medium with 1% pectin for 20 h shaking at 30^o^. Viscosity was measured by dropping a screwcap Eppendorf tube filled with glass beads (weighing 4.4 g) cap side down directly into the test tube, which was placed at a 45^o^ angle. The time for the Eppendorf tube to reach the bottom was recorded for each strain. Viscosity was averaged across three biological replicates and error was determined by standard deviation. The breakdown of pectin in mandarin oranges was tested by growing the indicated strains for 24 h in 200 mL YPGAL liquid cultures shaking at 30^o^. Cells were spun down by centrifugation and cell supernatants were harvested. Mandarins were purchased commercially from Sunrays (https://sunraysfruits.com/products/mandarins/). The mandarin was sterilely peeled and wedges were separated. One mandarin wedge and one piece of peel was placed in 50 mL supernatants of indicated strains and left at 30^o^ for 24 h. Wedges and peels were removed and placed under a gentle stream of water and washed. Images before and after treatment were captured using an iPhone 13 (Apple).

### Two-hybrid analysis

Two-hybrid analysis (216) was performed to identify protein interactions as described in (86, 217). The assay was done by transforming two-hybrid constructs into the yeast strain background PJ69-4a using the pGAD-C1–pGBDU-C1 system described in reference 218. Briefly, cells were spotted onto SD–Ura–Leu to maintain plasmid selection and to act as a control for growth. Cells were also spotted onto SD–Ura–Leu–His to assess the *LYS2::GAL1-HIS3* growth reporter. Positive control of a Cdc42p-Bem4p interaction is described in (83, 86, 219). Plasmid constructs used here are described in reference 86 for *RAS2*, *RAS2^G19V^*, *CDC24*, *STE11*, *STE50*, *KSS1*, *STE7*; in reference 83 for *BEM4, CDC42*, *BEM1*; in reference 36 for *OPY2*-tail; and in reference 217 for *STE20*.

## Data Availability

All data are in the paper and/or supporting information files.

## SUPPLEMENTAL MATERIAL

Supplemental material msphere.00284-23-s0001.pdf Fig. S1 to S9 and Table S1 and Table S2.

ASM does not own the copyrights to Supplemental Material that may be linked to, or accessed through, an article. The authors have granted ASM a non-exclusive, world-wide license to publish the Supplemental Material files. Please contact the corresponding author directly for reuse.
