# Supplementary material for: Ecological inducers of the yeast filamentous growth pathway reveal environment-dependent roles for pathway components
Source: mSphere. 2023 Sep 21;8(5):e00284-23. doi: 10.1128/msphere.00284-23 (PMC10597418; doi:10.1128/msphere.00284-23)
Supplement: Supplemental material — Fig. S1 to S9 and Table S1 and Table S2. [file msphere.00284-23-s0001.pdf]

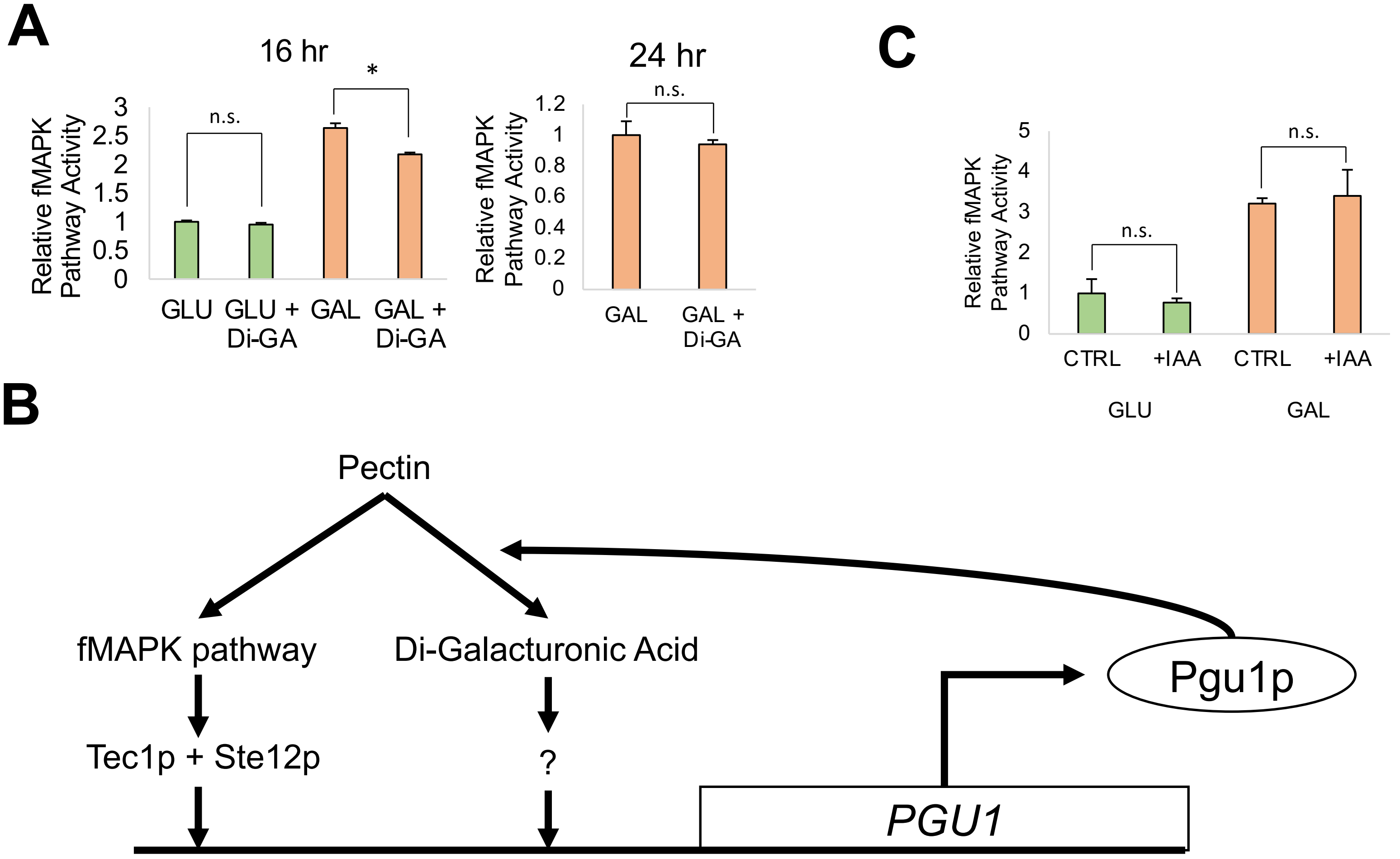

Figure S1. fMAPK pathway activity for galacturonic acid, di-galacturonic acid, and IAA. ß-galactosidase (*FRE-lacZ*) assays were performed in wild-type (PC313) cells. **A)** Cells were grown in 5 mL synthetic media with indicated carbon source after 16 h (left) or 24 h (right) and +3 mg of di-galacturonic acid as indicated. Average relative fMAPK pathway activity across at least 3 replicates is reported, with GLU values set to 1 (left) or GAL values set to 1 (right). Error bars represent standard deviation. Asterisk, p-value < 0.05 by Student's t-test for the indicated comparisons. **B)** Model of the regulation of *PGU1* expression. **C)** Cells were grown in 5 mL synthetic media with the indicated carbon source after 20 h and +0.1 mg of IAA dissolved in NaOH as indicated. For the control (CTRL), an equal amount of NaOH alone was added to the media. Average relative fMAPK pathway activity across at least 3 replicates is reported, with CTRL values in GLU set to 1. Error bars represent standard deviation. Asterisk, p-value < 0.05 by Student's t-test for indicated comparisons.

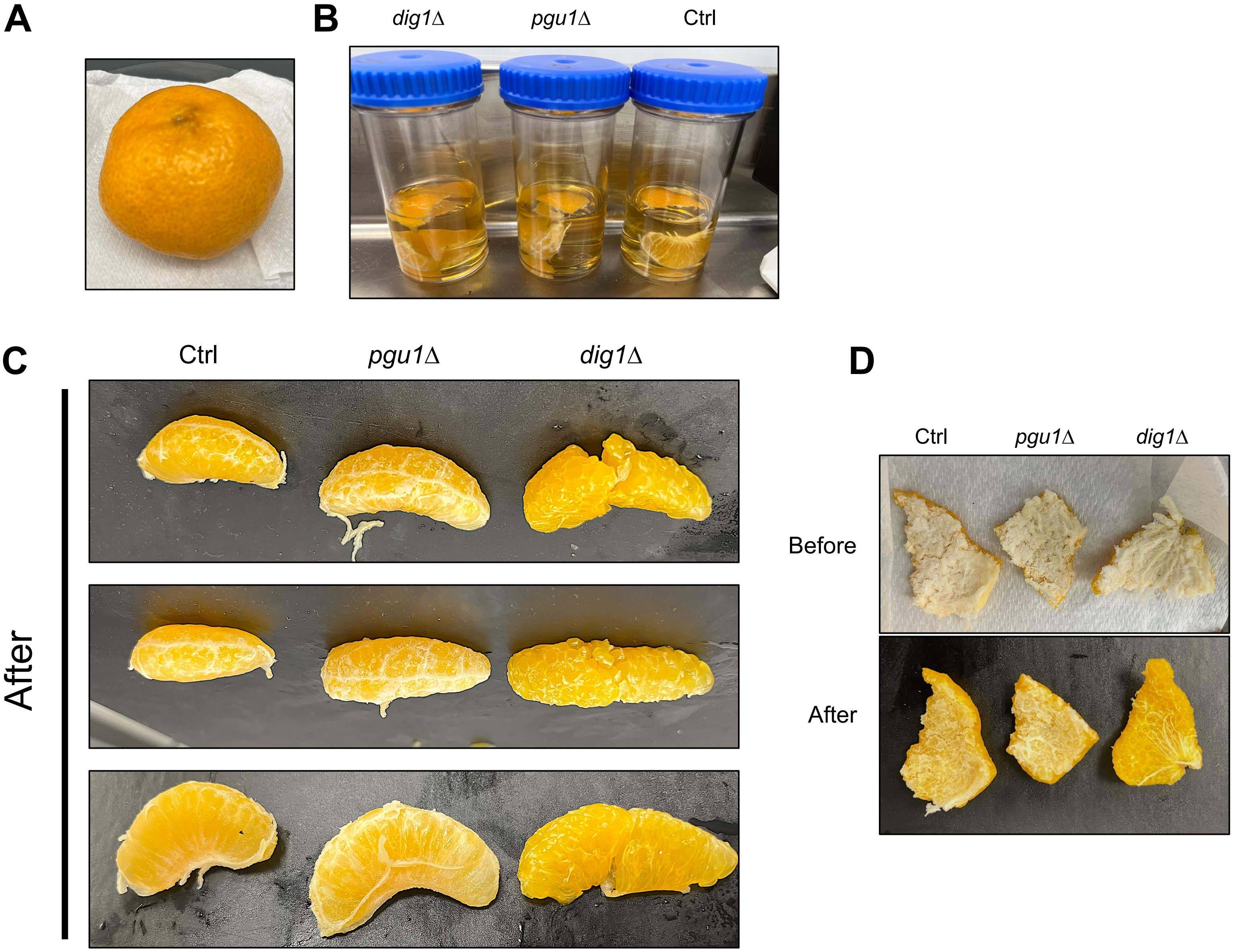

Figure S2. Examples of mandarin orange experiments. Mandarin orange wedges were submerged for 24 h in supernatants of *pgu1*Δ (PC7833) and *dig1*Δ (PC7676) mutants. Supernatants were derived from 24 h cultures in YPGAL. Ctrl, YPGAL media with no cells added. **A)** Image of mandarin orange. **B)** Image of mandarin wedges and peels submerged in cell supernatants at time zero. **C)** Images of wedges after submersion after 24 h. Three examples are shown. **D)** Images of peels before (top) and after (bottom) 24 h incubation with cultured supernatants.

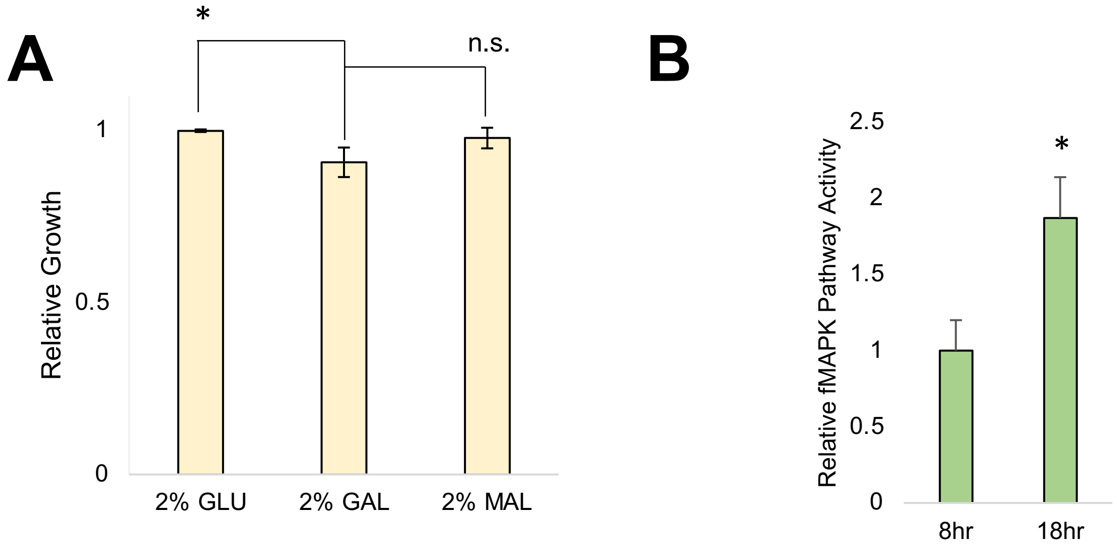

Figure S3. Glucose depletion stimulates the fMAPK pathway. A) Relative growth of wild-type cells (PC313) grown in 5 mL of synthetic media with indicated carbon source for 24 h. Average relative growth determined by OD_{600} across at least 3 replicates is reported, with GLU values set to 1. Error bars represent standard deviation. Asterisk, p-value < 0.05 by Student's t-test compared to 2% GAL. **B)** ß-galactosidase (*FRE-lacZ*) assays. Wild-type cells (PC313) were grown in 5 mL synthetic media with glucose for 8 h or 18 h. Average relative fMAPK pathway activity of at least 3 replicates are reported with 8 h GLU values set to 1. Error bars represent standard deviation. Asterisk, p-value < 0.05 by Student's t-test compared to 8 h GLU values.

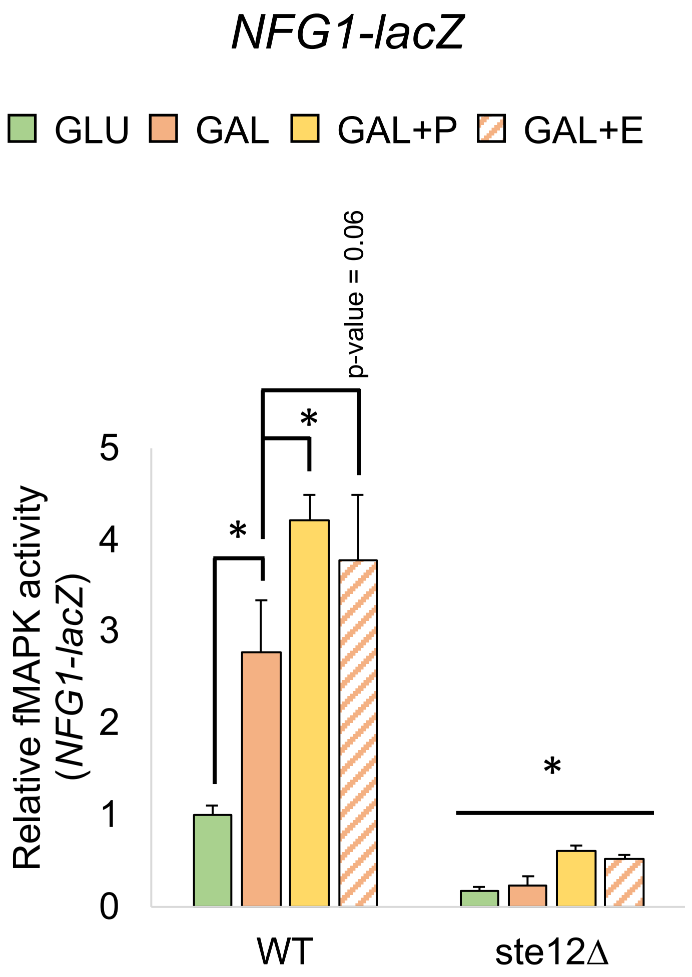

Figure S4. fMAPK pathway activity measured by the *NFG1-lacZ* reporter. ß-galactosidase (*NFG1-lacZ*) assays. Wild-type (PC586) cells and the *ste12*Δ (PC2184) mutant were grown in 2 mL synthetic media with indicated carbon source for 17 h. +P = + 1% pectin. +E = +3.85% ethanol. Average relative fMAPK pathway activity across at least 3 replicates is reported, with wild-type GLU values set to 1. Error bars represent standard deviation. Asterisk for wild-type values, p-value < 0.05 by Student's t-test compared to indicated condition. Asterisk for *ste12*Δ values, p-value < 0.05 by Student's t-test compared to wild-type values from same condition.

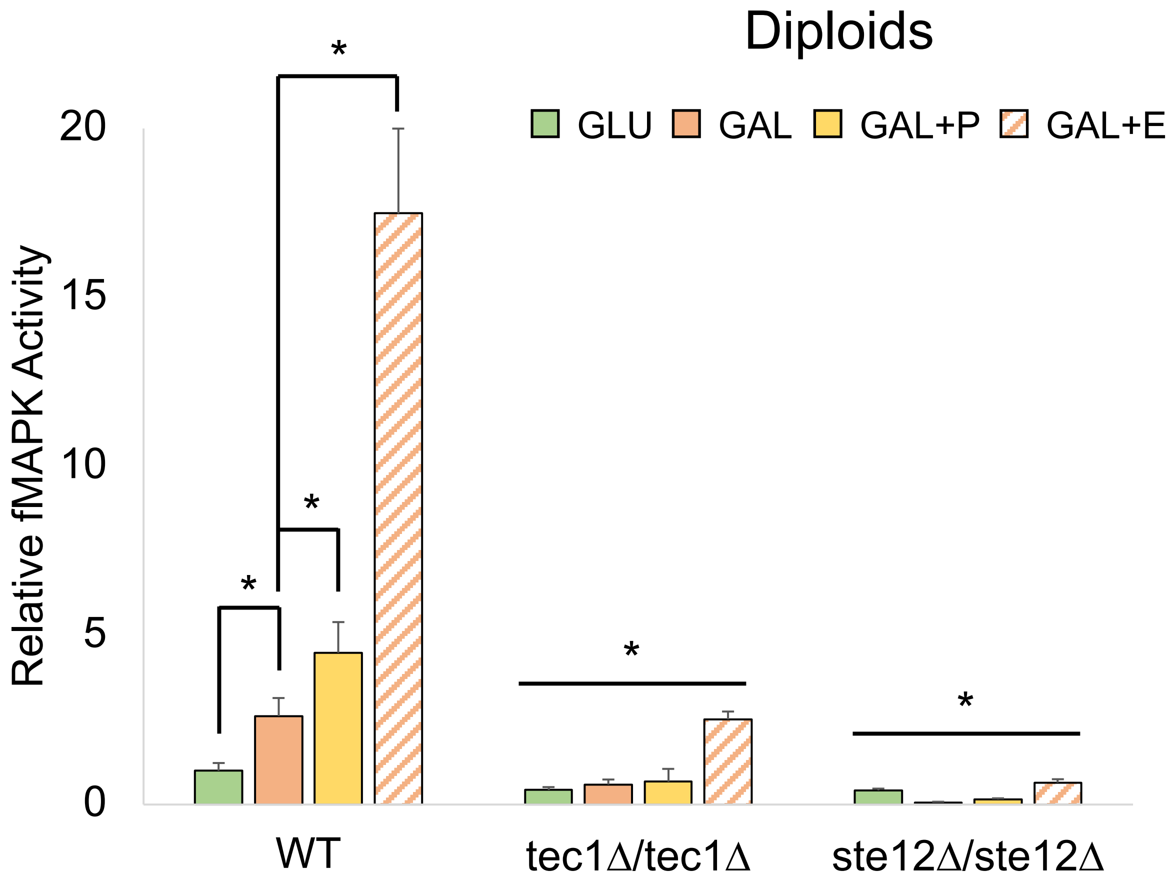

Figure S5. fMAPK pathway activity in diploids. ß-galactosidase (*FRE-lacZ*) assays. Wild-type MAT**a**/MAT*α* (PC344) cells and the *tec1Δ/tec1Δ* (PC7784) and *ste12Δ/ste12Δ* (PC7884) mutants were grown in 2 mL synthetic media with indicated carbon source for 17 h. +P = + 1% pectin. +E = +3.85% ethanol. Average relative fMAPK pathway activity across at least 3 replicates is reported, with wild-type GLU values set to 1. Error bars represent standard deviation. Asterisk for wild-type values, p-value < 0.05 by Student's t-test compared to indicated condition. Asterisk for mutant values, p-value < 0.05 by Student's t-test compared to wild-type values from same condition.

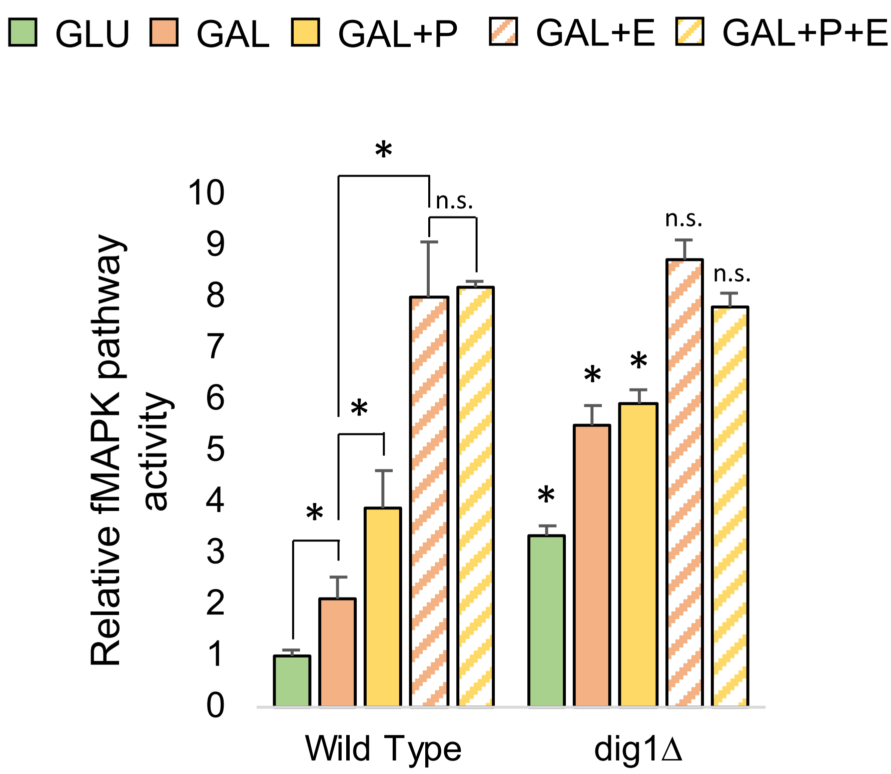

Figure S6. Combinatorial effects of inducers on fMAPK pathway activity. ß-galactosidase (*FRE-lacZ*) assays. Wild-type (PC313) cells and the *dig1*Δ (PC7676) mutant were grown in 2 mL synthetic media with indicated carbon source for 24 h. +P = + 1% pectin. +E = +3.85% ethanol. Pectin with ethanol was not tested because cells failed to grow due to a lack of carbon source, combined with the growth inhibition caused by ethanol. Average relative fMAPK pathway activity across at least 3 replicates is reported, with wild-type GLU values set to 1. Error bars represent standard deviation. Asterisk for wild-type values, p-value < 0.05 by Student's t-test compared to indicated condition. Asterisk for *dig1*Δ values, p-value < 0.05 by Student's t-test compared to wild-type values from same condition.

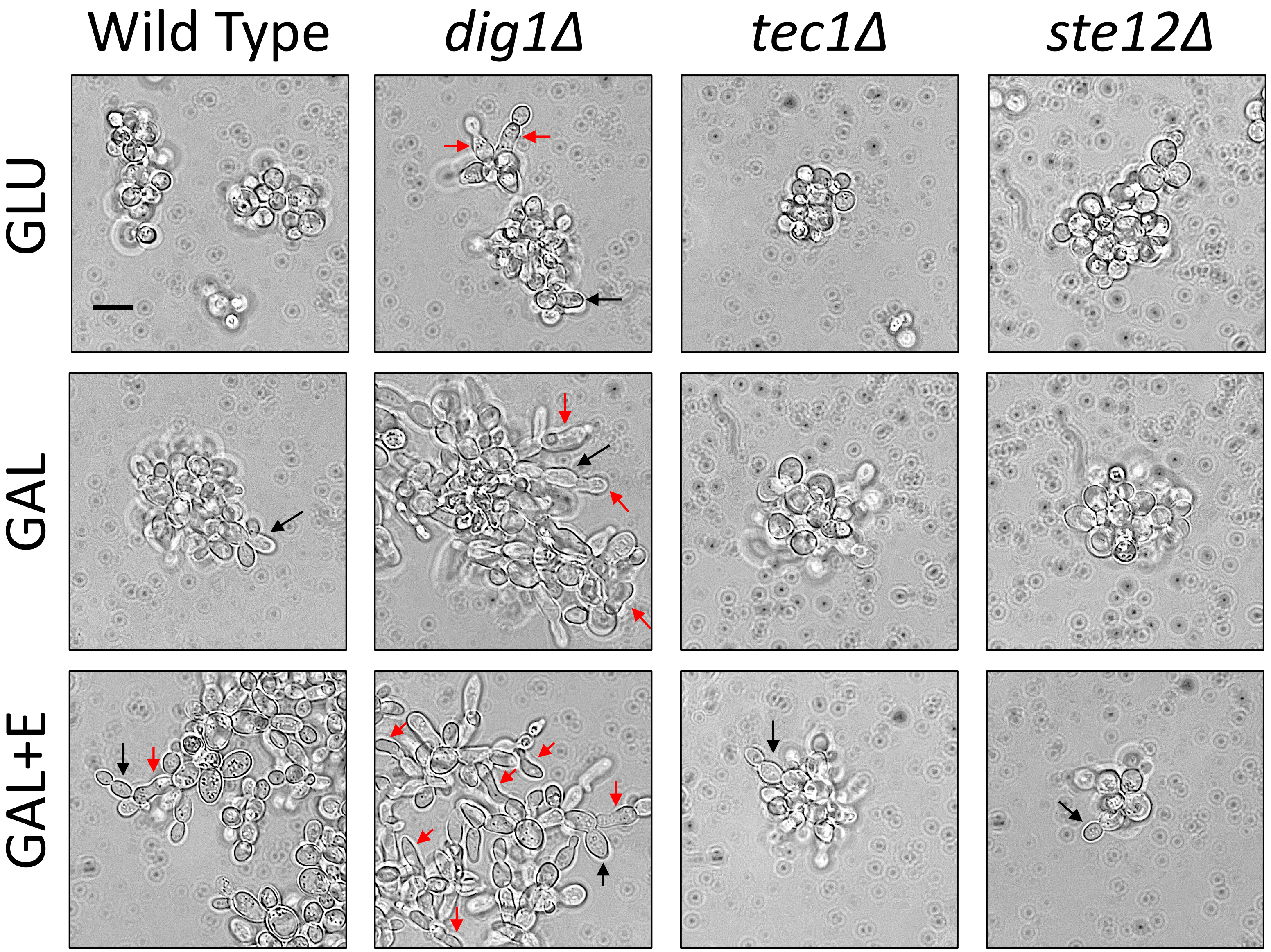

Figure S7. Additional images for % filamentous growth assay. Wild-type (PC313) cells and the *ste12*Δ (PC5651), *tec1*Δ (PC7675) and *dig1*Δ (PC7676) mutants were examined by microscopy in indicated media and imaged. Microscopy images taken at 100X in indicated media. Bar, 10 um. Black arrow, filamentous cell. Red arrow, cell displaying aberrant morphology.

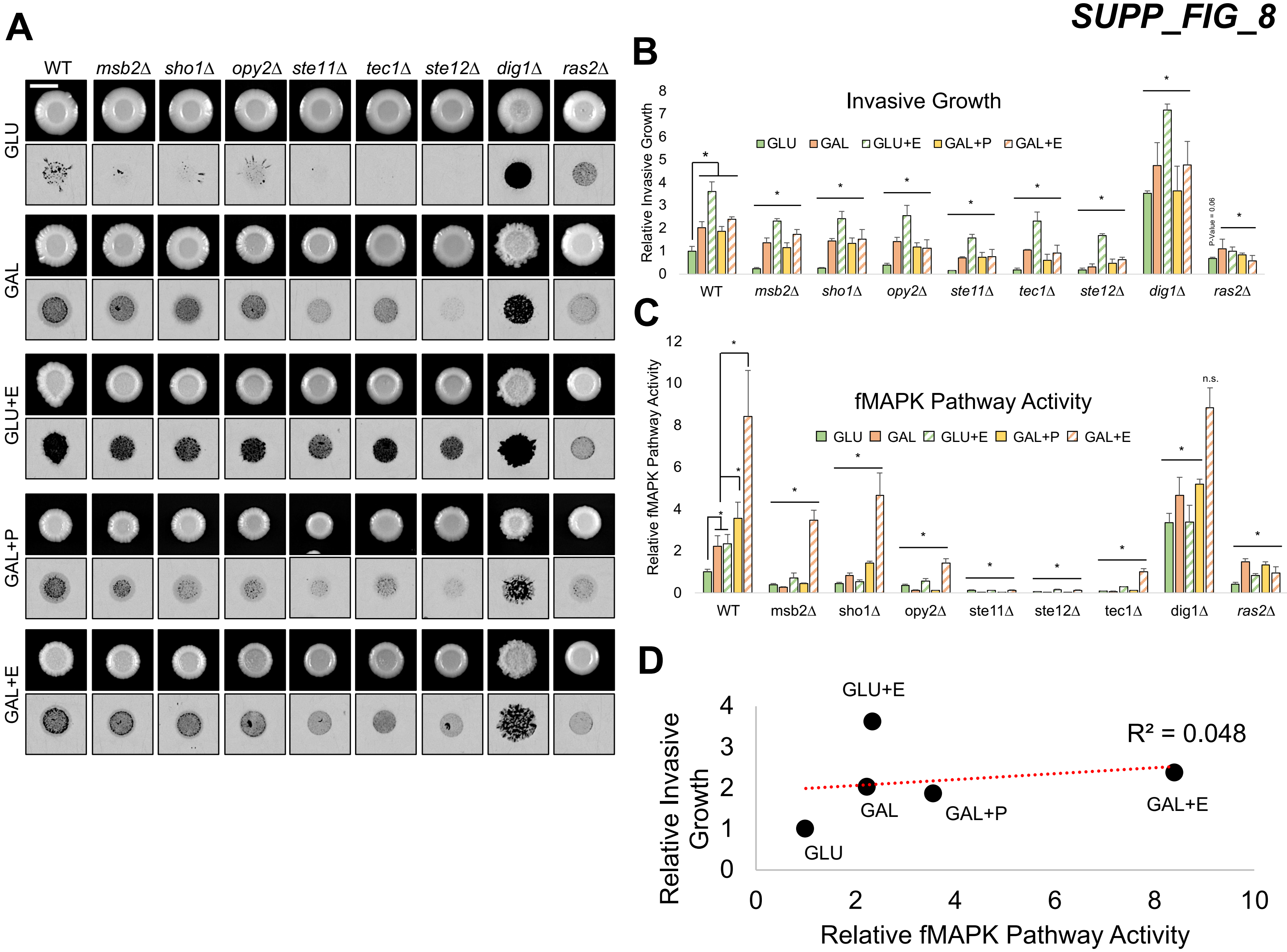

Figure S8. Full analysis of the plate-washing assay. A) Plate-washing assay. Wild-type (PC313) cells and the *msb2*Δ (PC961), *opy2*Δ (PC3894), *sho1*Δ (PC5026), *ste11*Δ (PC5024), *ste12*Δ (PC5651), *tec1*Δ (PC7675), *dig1*Δ (PC7676), and *ras2*Δ (PC6222) mutants were spotted for 5 d on the indicated medium. Top row, before wash. Bottom row, inverted images of invasive scars after wash. Bar, 0.5 cm. **B)** Quantitation of the data shown in panel S5A. Average relative invasion across at least 3 replicates is reported, with wild-type values in GLU set to 1. Error bars represent the standard deviation. Asterisk for wild-type values, p-value < 0.05 by Student's t-test compared to indicated condition. Asterisk for mutants' values, p-value < 0.05 by Student's t-test compared to wild-type values from same condition. **C)** The same fMAPK pathway activity data from **Fig 3A** for comparison to invasive growth. **D)** fMAPK pathway activity compared to invasive growth for wild-type cells on indicated media reveals no correlation, $R^2 = 0.048$. Data values are repeated from panels B and C.

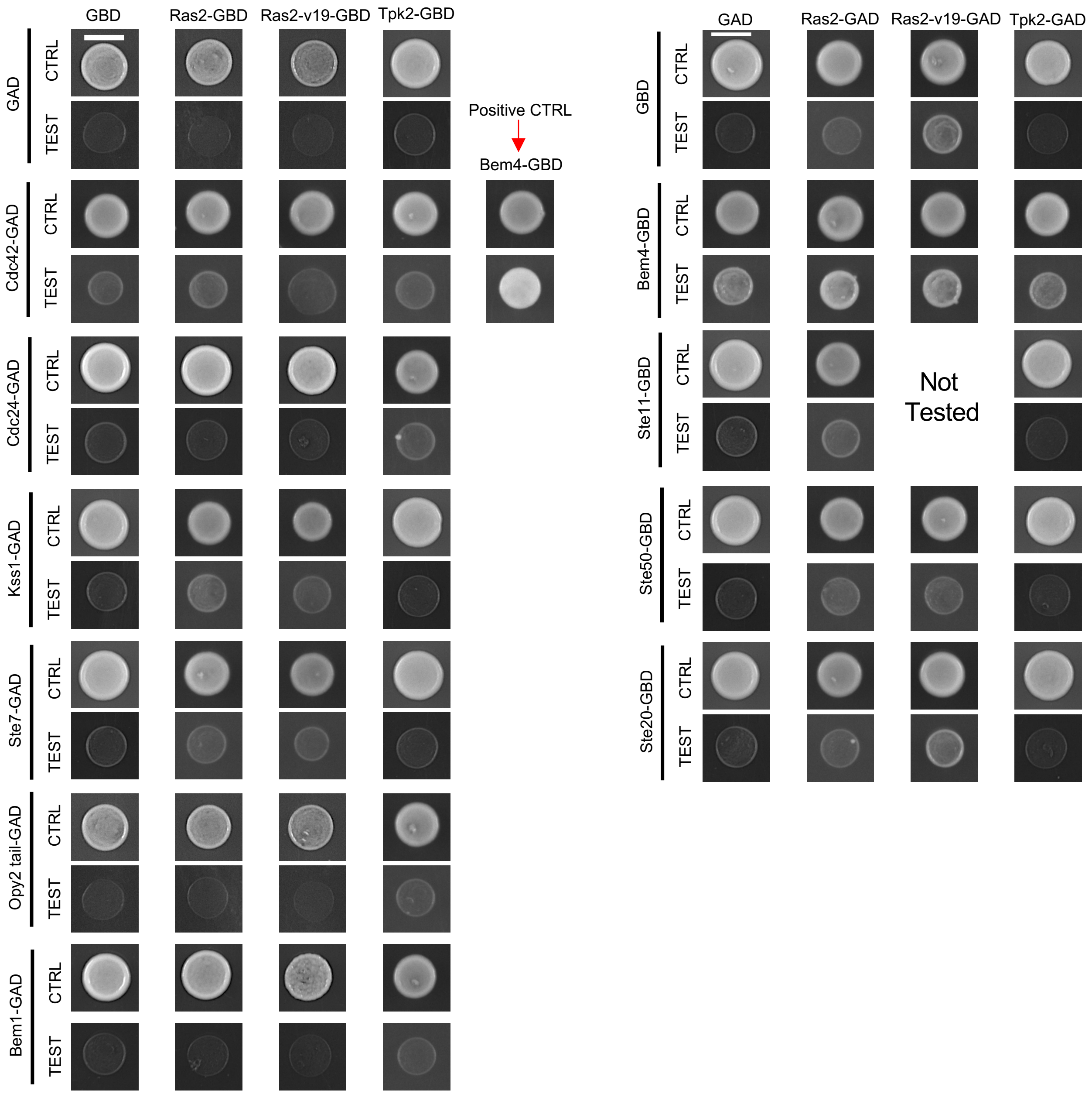

Figure S9. Two-hybrid analysis. Y2H analysis between Ras2p, a hyperactive version (Ras2^{G12V}), and the PKA pathway kinase, Tpk2p, with components of the fMAPK pathway. Red arrow, interaction between Bem4p and Cdc42p (*86*) was used as a positive control. CTRL, SD-Ura-Leu media supplemented with HIS. TEST, SD-Ura-Leu media not supplemented with HIS. Growth on TEST plate represents an interaction.

Table S1. Yeast strains used in this study.

Strain (PC#)	Description	Haploid/Diploid	Reference
313	*MAT***a** *ura3-52*	Haploid	(Liu et al., 1993)
586	*MAT***a** *ura3-52 leu2Δ*	Haploid	(Cullen et al., 2004)
961	*MAT***a** *ura3-52 msb2Δ::KanMX6*	Haploid	(Cullen et al., 2004)
2184	*MAT***a** *ura3-52 leu2Δ ste12Δ*	Haploid	(Pitoniak et al., 2009)
3343	*MAT***a** *ura3-52 bem4* Δ::HYG	Haploid	(Pitoniak et al., 2015)
3894	*MAT***a** *ura3-52 opy2Δ::* NAT	Haploid	(Karunanithi et al., 2012)
4982	*MAT***a** *ura3-52 ste50Δ::* NAT	Haploid	(Karunanithi et al., 2012)
4988*	*MAT***a** *trpl-901 leu2-3,112 ura3-52 his3-200 ga14A ga18OA LYS2::GALI-HIS3 GAL2-ADE2 met2::GAL7-lacZ*	Haploid	(James et al., 1996)
5024	*MAT***a** *ura3-52 ste11Δ::* NAT	Haploid	(Pitoniak et al., 2015)
5026	*MAT***a** *ura3-52 sho1Δ::* NAT	Haploid	(Pitoniak et al., 2015)
5035	*MAT***a** *ura3-52 pbs2Δ::* NAT	Haploid	(Pitoniak et al., 2015)
5187	*MAT***a** *ura3-52 mig1Δ::* NAT *mig2* Δ::HYG	Haploid	(Karunanithi et al., 2012)
5651	*MAT***a** *ura3-52 ste12Δ::* NAT	Haploid	(Chavel et al., 2014)
6048	*MAT***a** *ura3-52 ire1::* NAT	Haploid	(Adhikari et al., 2015)
6222	*MAT***a** *ura3-52 ras2::* HYG	Haploid	(Chavel et al., 2014)
7673	*MAT***a** *ura3-52 rim101* Δ::NAT	Haploid	(Vandermeulen and Cullen, 2022)
7674	*MAT***a** *ura3-52 opi1* Δ::NAT	Haploid	(Vandermeulen and Cullen, 2022)
7675	*MAT***a** *ura3-52 tec1* Δ::NAT	Haploid	(Vandermeulen and Cullen, 2022)
7676	*MAT***a** *ura3-52 dig1* Δ::NAT	Haploid	(Vandermeulen and Cullen, 2022)
7677	*MAT***a** *ura3-52 rtg3* Δ::NAT	Haploid	(Vandermeulen and Cullen, 2022)
7833	*MAT***a** *ura3-52 pgu1* Δ::NAT	Haploid	This study
7844	*MAT***a** *ura3-52 gal7* Δ::HYG	Haploid	This study
7845	*MAT***a** *ura3-52 gal4* Δ::HYG	Haploid	This study
7846	*MAT***a** *ura3-52 gal10* Δ::HYG	Haploid	This study
7849	*MAT***a** *ura3-52 gal3* Δ::HYG	Haploid	This study
7865	*MAT***a** *ura3-52 flo8* Δ::NAT	Haploid	This study
7869	*MAT***a** *ura3-52 tpk3* Δ::NAT	Haploid	This study
7870	*MAT***a** *ura3-52 bcy1* Δ::NAT	Haploid	This study
7871	*MAT***a** *ura3-52 ste20* Δ::NAT	Haploid	This study
7872	*MAT***a** *ura3-52 pde2* Δ::NAT	Haploid	This study
7873	*MAT***a** *ura3-52 phd1* Δ::NAT	Haploid	This study
7874	*MAT***a** *ura3-52 tpk2* Δ::NAT	Haploid	This study
344	*MAT*a/*α ura3-52/ura3-52*	Diploid	(Cullen et al., 2004)
7784	*MAT*a/*α ura3-52/ura3-52 tec1Δ/tec1Δ*	Diploid	This study
7884	*MAT*a/*α ura3-52/ura3-52 ste12Δ/ste12Δ*	Diploid	This study

*in PJ69-4a background

Table S2. Primers used in this study to generate deletion mutants.

Gene	Forward Primer - 5' to 3'	Reverse Primer - 5' to 3'	Reference
TPK2	ATGGAATTCGTTGCAGAAAGGGCTCAGCCAGTTGGTCAAATAGGCCACTAGTGGATCTG	TTAGAAATCTTGAAAGTATTCAGCATATGGATCATCGCCTTAGCTGAAGCTTCGTACGC	(Chavel *et al.*, 2010)
PHD1	ATGTACCATGTTCCTGAAATGAGGCTACATTACCCCCTGGTGAACATAGGCCACTAGTGGATCTG	TTATGATAATTCATTTTTTGCTCTACTTGTTTGGGCCTCAGTATCAGCTGAAGCTTCGTACGC	(Chavel *et al.*, 2014)
PDE2	ATGTCCACCCTTTTTCTGATTGGAATACACGAGATTGAGAAATCTATAGGCCACTAGTGGATCTG	CTATTGTGGTTTCTTGTGTTTCATCCAGTATTCTTTATTGATTTTAGCTGAAGCTTCGTACGC	(Chavel *et al.*, 2010)
TPK3	ATGTATGTTGATCCGATGAACAACAATGAAATCAGGAAATTAAGCAATAGGCCACTAGTGGATCTG	AAAATTCTTTCATTAAATCCATATATGGATCCTCCCCTTGAATTCAGCTGAAGCTTCGTACGC	This study
BCY1	ATGGTATCTTCTTTGCCCAAGGAATCGCAAGCCGAATTGCAACTGTATAGGCCACTAGTGGATCTG	GTCTTGTAGGATCATTGAGCTTTAATACGTCTACTGCAGGACCCAGAGCTGAAGCTTCGTACGC	This study
STE20	ATGAGCAATGATCCATCTGCTGTATCGGAACTACCAGACAAGGACAATAGGCCACTAGTGGATCTG	TTACTTTTGTTTATCATCTTCAGTTACGTCCAAATTTACGGTTTCTAGCTGAAGCTTCGTACGC	This study
PGU1	TTCACAATTATTCCATACAGGTATAAAAACGCACAGAACTTCATAGGCCACTAGTGGATCTG	AGCATAAATTGCCGAACAAATACCAATATTTTCCTGCATCTTAGCTGAAGCTTCGTACGC	This study
GAL7	ATGACTGCTGAAGAATTTGATTTTTCTAGCCATTCCCATAGACGTTATAGGCCACTAGTGGATCTG	ATAATGAATCTGACCATCTAAATTTCTTAGTTTTTCAGCAGCTTGTAGCTGAAGCTTCGTACGC	This study
GAL10	ATGACAGCTCAGTTACAAAGTGAAAGTACTTCTAAAATTGTTTTGGATAGGCCACTAGTGGATCTG	GAAAATCTGTAGACAATCTTGGACCCGTAAGTTTCACCGTTTTTCAAGCTGAAGCTTCGTACGC	This study
GAL4	ATGAAGCTACTGTCTTCTATCGAACAAGCATGCGATATTTGCCGACATAGGCCACTAGTGGATCTG	TTTGGGTTTGGTGGGGTATCTTCATCATCGAATAGATAGTTATATAAGCTGAAGCTTCGTACGC	This study
GAL3	ATGAATACAAACGTTCCAATATTCAGTTCTCCGGTCAGAGATTTACATAGGCCACTAGTGGATCTG	TCGTACAAACAAGTACCCAAGGCAGGCTTCGAAACTATAATTGCGTAGCTGAAGCTTCGTACGC	This study
FLO8	ATGAGTTATAAAGTGAATAGTTCGTATCCAGATTCAATTCCTCCCAATAGGCCACTAGTGGATCTG	AATAAAATTGAAATCATTTTCATTTGTATCCAGTAAATTGAGATCAGCTGAAGCTTCGTACGC	This study
TEC1 by CRISPR	TGCCAGATTCCTCCCATATCGACAAGAAGAATAATCCACCTATTTCAACAATTCTGATAC	GCGTATTTATGTACGAGATGTATGTATGTAGGTTAAACAGGTATCAGAATTGTTGAAATA<br>CAATCATATCTGTGACGGTTCTTACTACGAAGATACGTATGCGTATTTATGTACGAGATG	This study
STE12 by CRISPR	AATTGTCTTGTTCACCAAGGATGAAAGTCCAAATAACCAATAGTAGAACAGAGGAAATCT	AATGAGCTCCACCTTCTTCTGACTGGACCACTGAACTTTTAAGATTTCCTCTGTTCTACT<br>GGTTTTTATCGGACCTTCGATTGGTATCTACCTCAAGTGAATGAGCTCCACCTTCTTCTG	This study
